# Cortistatin deficiency reveals a dysfunctional brain endothelium with impaired gene pathways, exacerbated immune activation, and disrupted barrier integrity

**DOI:** 10.1186/s12974-023-02908-5

**Published:** 2023-10-04

**Authors:** Julia Castillo-González, José Luis Ruiz, Ignacio Serrano-Martínez, Irene Forte-Lago, Ana Ubago-Rodriguez, Marta Caro, Jesús Miguel Pérez-Gómez, Alejandro Benítez-Troncoso, Eduardo Andrés-León, Macarena Sánchez-Navarro, Raúl M. Luque, Elena González-Rey

**Affiliations:** 1 Institute of Parasitology and Biomedicine Lopez-Neyra (IPBLN), CSIC, PT Salud, 18016 Granada, Spain; 2 Maimonides Biomedical Research Institute of Cordoba (IMIBIC), 14004 Cordoba, Spain; 3 Department of Cell Biology, Physiology, and Immunology, University of Cordoba, 14004 Cordoba, Spain; 4 Reina Sofia University Hospital (HURS), 14004 Cordoba, Spain; 5 CIBER Physiopathology of Obesity and Nutrition (CIBERobn), 14004 Cordoba, Spain

**Keywords:** Blood–brain barrier, Cortistatin, Tight-junctions, Brain endothelium transcriptome, Oxygen–glucose deprivation, Ischemia, Endothelial immune activation

## Abstract

**Background:**

Brain activity governing cognition and behaviour depends on the fine-tuned microenvironment provided by a tightly controlled blood–brain barrier (BBB). Brain endothelium dysfunction is a hallmark of BBB breakdown in most neurodegenerative/neuroinflammatory disorders. Therefore, the identification of new endogenous molecules involved in endothelial cell disruption is essential to better understand BBB dynamics. Cortistatin is a neuroimmune mediator with anti-inflammatory and neuroprotective properties that exerts beneficial effects on the peripheral endothelium. However, its role in the healthy and injured brain endothelium remains to be evaluated. Herein, this study aimed to investigate the potential function of endogenous and therapeutic cortistatin in regulating brain endothelium dysfunction in a neuroinflammatory/neurodegenerative environment.

**Methods:**

Wild-type and cortistatin-deficient murine brain endothelium and human cells were used for an in vitro barrier model, where a simulated ischemia-like environment was mimicked. Endothelial permeability, junction integrity, and immune response in the presence and absence of cortistatin were evaluated using different size tracers, immunofluorescence labelling, qPCR, and ELISA. Cortistatin molecular mechanisms underlying brain endothelium dynamics were assessed by RNA-sequencing analysis. Cortistatin role in BBB leakage was evaluated in adult mice injected with LPS.

**Results:**

The endogenous lack of cortistatin predisposes endothelium weakening with increased permeability, tight-junctions breakdown, and dysregulated immune activity. We demonstrated that both damaged and uninjured brain endothelial cells isolated from cortistatin-deficient mice, present a dysregulated and/or deactivated genetic programming. These pathways, related to basic physiology but also crucial for the repair after damage (e.g., extracellular matrix remodelling, angiogenesis, response to oxygen, signalling, and metabolites transport), are dysfunctional and make brain endothelial barrier lacking cortistatin non-responsive to any further injury. Treatment with cortistatin reversed in vitro hyperpermeability, tight-junctions disruption, inflammatory response, and reduced in vivo BBB leakage.

**Conclusions:**

The neuropeptide cortistatin has a key role in the physiology of the cerebral microvasculature and its presence is crucial to develop a canonical balanced response to damage. The reparative effects of cortistatin in the brain endothelium were accompanied by the modulation of the immune function and the rescue of barrier integrity. Cortistatin-based therapies could emerge as a novel pleiotropic strategy to ameliorate neuroinflammatory/neurodegenerative disorders with disrupted BBB.

**Supplementary Information:**

The online version contains supplementary material available at 10.1186/s12974-023-02908-5.

## Introduction

The blood–brain barrier (BBB) is a highly dynamic interface between the central nervous system (CNS) and the peripheral circulation [1]. BBB is comprised of brain endothelial cells (BECs) surrounded by astrocyte end-feet and pericytes, all of them embedded in an extracellular matrix network [2]. The function of the BBB is intricately controlled as it provides a tight regulation of ions and nutrient influx, restricts the transport of harmful agents, and selectively limits the traffic of immune cells and inflammatory mediators [1]. Brain endothelial cells show unique features compared to other endothelial peripheral cells that allow this fine regulation. Among them, it is the presence of tight-junction (TJ) and adherens-junction (AJ) proteins, which attach adjacent endothelial cells and connect to cytoskeletal proteins, such as actin, thereby maintaining the structural integrity of the endothelium [3]. In addition, endothelial cells exhibit a low rate of pinocytosis, present several transporters that facilitate nutrient uptake and waste elimination, and express low levels of leukocyte adhesion molecules [1]. BBB disruption is a key feature of most neuroinflammatory/neurodegenerative disorders, such as Parkinson’s and Alzheimer’s disease, or brain ischemia. This provokes an imbalance in the transport of and/or permeability to proteins, inflammatory cells, or pathogens, leading to brain homeostasis failure, glial activation, and neuronal dysfunction [4]. Importantly, BECs dysfunction is sufficient to promote BBB breakdown, and this is mainly attributed to the loss of junction integrity [3]. Therefore, the identification of new endogenous molecules that target endothelial cell dysfunction seems essential to better understand and promote BBB stability and maintenance.

Cortistatin is a cyclic neuropeptide first discovered in the brain cortex and hippocampus but widely distributed in the immune system [5, 6]. Highly homologous to somatostatin (SST), cortistatin can bind to somatostatin receptors (SSTR1–5) sharing functional properties with SST. However, cortistatin displays unique functions in the nervous system and peripheral tissues [7, 8] by interacting with other receptors different from those of SST, including the ghrelin receptor (GHSR1α), the Mas gene-related receptor X-2 (MRGPRX2), and a still unidentified cortistatin-selective receptor. In fact, cortistatin has been described as a potent anti-inflammatory molecule that protects against the development and progression of several inflammatory and autoimmune disorders (reviewed in [5]). Specifically, multiple studies have reported that cortistatin treatment exerts immunomodulatory and neuroprotective effects in models of neuroinflammatory/neuroimmune injury, such as excitotoxicity [9], meningoencephalitis [10], multiple sclerosis [11], or neuropathic pain [12]. In all of them, cortistatin reduced inflammatory mediators and modulated glial cell function while maintaining their tissue-surveillance activities. Interestingly, the lack of cortistatin has been reported to cause an exacerbated pro-inflammatory basal state in the CNS and periphery, accompanied by decreased expression of neurotrophic factors [5, 11]. Moreover, cortistatin plays a key role in peripheral vascular function, as it has been shown that the arterial endothelium expresses cortistatin and its receptors, especially in response to injury, and that cortistatin impairs the binding of immune cells to the peripheral endothelium [13–15].

Hence, the anti-inflammatory, immunomodulatory, and neuroprotective properties of cortistatin, together with its critical function in the dynamics and activation of immune cell populations in the periphery and in the glial niche, highlight the key role of cortistatin in brain-injury disorders. Taking into account the relevance of the brain barrier for CNS homeostasis and the protective properties of cortistatin in the peripheral endothelium, an effect of cortistatin in regulating BBB physiology and its breakdown would be expected. However, nothing is known about the role of this neuropeptide in either the brain endothelium or the integrity of the BBB. Therefore, in this study we aimed to investigate the potential function of cortistatin in regulating brain endothelium dysfunction in a neuroinflammatory/neurodegenerative environment and to elucidate the relevance of the absence of cortistatin in BBB dynamics.

## Materials and methods

### Animals

All procedures described in this study were approved by the Animal Care and Use Board and the Ethical Committee of the Spanish National Research Council (Animal Care Unit Committee IPBLN-CSIC # protocol CEEA OCT/2017.EGR), in accordance with the guidelines from Directive 2010/63/EU of the European Parliament on the protection of animals used for scientific purposes. Transgenic mice lacking the cortistatin gene (*Cort*^−/−^) were a kind gift from Dr. Luis de Lecea (Stanford University, La Jolla, CA, USA). They were generated and also maintained as hemizygotes (*Cort*^+/^^−^) in a C57BL/6J background as previously described [7, 12] and in Additional file 1.

### Cell culture

#### Preparation of b.End5 cells

The mouse brain endothelioma cell line b.End5 (Sigma-Aldrich, #96091930) was used as a BBB in vitro model [16]. b.End5 cells were incubated as reported in Additional file 1. Before procedures, adherent cells were rinsed in 1 × phosphate buffer solution (PBS) and detached by adding 0.1% trypsin–EDTA, followed by the addition of an equal volume of fresh medium. The cell suspensions were centrifuged for 5 min at 150 rcf. The supernatant was then removed and cells were resuspended in fresh medium, counted, and allowed to grow at 37 ºC and 5% CO_2_ in a humidified incubator.

#### Isolation of mouse brain endothelial cells (BECs)

To isolate endothelial cells (ECs) from mouse brain microvasculature, we followed a dissociation protocol [17] with some modifications. Additional details can be found in Additional file 1. Prior to cell seeding for experiments, adherent BECs were trypsinized (0.05% trypsin–EDTA), centrifuged for 5 min at 290 rcf, and brought to suspension as described above for b.End5 cells.

#### Human BBB model

A human BBB model was generated by differentiating cord blood-derived hematopoietic cells into ECs, followed by the induction of BBB properties by co-culturing them with pericytes (see Additional file 1), as previously described [18]. After 6–8 days of co-culture, ECs acquired BBB properties (therefore, named human brain-like endothelial cells, hBLECs), and were used in permeability assays.

### Brain endothelium barrier model

To establish the brain endothelium barrier model, mouse ECs (b.End5, BECs) (5 × 10^4^ cells/well) were seeded on the top of a PET transwell insert (0.33 cm^2^, 8 µm pore size, Corning) previously coated with rat collagen-I (50 μg/ml) and fibronectin (20 μg/ml, Invitrogen). For hBLECs, inserts with cells from co-cultures were placed into new wells without pericytes. Cells were grown to confluence in their respective culture media. We considered that endothelial cell barrier integrity in murine monocultures was established when the transendothelial electrical resistance (EVOM^2^ Epithelial Voltohmmeter, World Precision Instruments) was above 200 Ω × cm^2^. TEER measurements under NX/NG conditions were evaluated in random transwells from each assay prior to any experimental procedure for b.End5 (550 ± 25.17 Ω × cm^2^), BECs isolated from *Cort*^+/+^ (585.7 ± 22.15 Ω × cm^2^), *Cort*^+/−^ (505.5 ± 27.78 Ω × cm^2^), and *Cort*^−/−^ (525 ± 25.28 Ω × cm^2^) mice. Human barrier integrity was also evaluated by measuring the permeability of Lucifer yellow (Pc < 15·10^–6^ cm/s) as described below and detailed in Additional file 1.

### Establishment of brain injury-like conditions

Mouse ECs were exposed to bacterial lipopolysaccharide (LPS) inflammation, glucose deprivation (GD), or oxygen–glucose deprivation and reoxygenation (OGD-R), while hBLECs were incubated in a hypoxic environment as well as in OGD-R conditions. For inflammatory damage, LPS from *E. coli* 055:B5 (10 μg/ml, Sigma) was added to the cells for 24 h. For GD conditions, b.End5 were washed twice and placed in glucose and serum-free b.End5 culture medium for 24 h. For OGD-R, mouse cells were placed in their respective glucose and serum-free culture media and incubated in a sealed hypoxic workstation (1% O_2_, 94% N_2_, 5% CO_2_, 37 ºC; In vivO_2_, Ruskin Technologies) for 4 h. Immediately after OGD, cultures were returned to normoxic (NX) and normoglycemic (NG) conditions (21% O_2_, 5.5 mM glucose), and incubated in 10% FBS medium for 20 h to mimic reperfusion. hBLECs were incubated under hypoxic conditions (HPX, 1% O_2_) for 4 h in normal sECM/5% FBS medium and then returned to control conditions for 20 h (HPX-R). For OGD, hBLECs were incubated in a hypoxic chamber for 6 h. Subsequently, cells were returned to NX conditions for 18 h. Unlike from hypoxia, OGD and OGD-R conditions were performed in endothelial cell medium in the absence of serum, glucose or other commercial growth factors. When needed, mouse cortistatin-29 or human cortistatin-17 (CST; Bachem) (100 nM) were added for 24 h immediately after LPS and GD, or for 20 h during reoxygenation in OGD-R or HPX-R conditions. For each cell type, controls were incubated simultaneously in normal medium in a NX/NG environment (21% O_2_, 5.5 mM glucose at 37 ºC).

### Endothelial permeability in vitro models

After LPS, GD, OGD-R or HPX-R incubations, Evans Blue-Albumin (Sigma, EBA, 67 kDa) and sodium fluorescein (Sigma, NaF, 376 Da) influx was determined as described [19] (details in Additional file 1). The permeability coefficient (Pc) of the tracer was expressed as cm/min of the tracer diffusing from the luminal to the abluminal side as Pc = (*C*_A_/*t*)*(1/*A*)*(*V*/*C*_L_). *C*_A_ is the tracer concentration in the abluminal side, *t* is the time (60 min), *A* is the total surface of the insert membrane, *V* is the final volume, and *C*_L_ is the initial known tracer concentration in the luminal side. To avoid inter-variability between assays, permeability was finally represented as the index (%) between the Pc of each well and the Pc of the control transwell (maximal influx of each tracer in an ECs-free coated insert).

### Immunocytochemistry

After exposure to insults, cultured mouse/human ECs were fixed with 4% paraformaldehyde (Sigma) for 10 min at RT. For claudin-5 staining, hBLECs were fixed with cold methanol and rinsed twice with cold PBS. Specific immunodetection was performed as described in Additional file 1. Cells from four different fields for each cell culture were imaged at 63 × magnification in a LEICA DMi8 S Platform Life cell microscope, selecting different region-of-interest (ROI) with the same exposure parameters. At least six independent experiments (cell cultures) were performed and a total of 25–50 selected ROIs were examined for each group. Image fluorescence analyses (mean grey value) were performed with ImageJ Fiji free software (https://fiji.sc).

### Determination of inflammatory factors and cortistatin

The concentration of inflammatory mediators and cortistatin in b.End5/BECs/hBLECs culture media was determined by ELISA assay. The amount of nitric oxide was estimated from the accumulation of nitrite by the Griess assay. Details can be found in Additional file 1.

### Transendothelial migration assay

Before migration experiments, immune cells were isolated from 8-week-old *Cort*^+/+^ mice. Macrophages and T cells were isolated as described in Additional file 1. Immune migration was evaluated in a BECs monolayer grown onto coated inserts of 24-well plates, as described above. 24 h before the assay, cells were activated with TNF-α (10 ng/ml, BD Pharmigen). On the day of the experiment, transwells inserts were transferred to a new 24-well culture plate containing 1% FBS medium in the bottom side supplemented with MCP-1 (50 μg/ml, Bionova) or IP-10 (50 μg/ml, Bionova) for macrophages and T cells, respectively. Immediately, immune cells (2 × 10^5^/transwell) were added to the upper side of the transwell in contact with BECs. When indicated, mouse cortistatin-29 (100 nM) was added to both sides of the transwell. After 24 h, migrated T cells were counted on the bottom side using a Neubauer chamber (VWR). Macrophages were identified at the bottom of the well after plate fixation and DAPI staining. As a positive control of migration, macrophages/T cells were incubated in a BECs-free coated transwell. The number of migrated cells was represented as the percentage of immune migrated cells vs the positive control.

### In vivo blood–brain barrier permeability assay

BBB integrity was analysed by evaluating Evans blue extravasation. For this, 1-year-old *Cort*^+*/*+^ and *Cort*^*−/−*^ mice were injected intraperitoneally with LPS from *E.coli* 055:B5 (6 mg/kg, Sigma) inducing mild neuroinflammation [20]. Immediately after LPS injection, 1 nmol of CST-29 (resuspended in PBS, Bachem) was intraperitoneally injected. 5 h after LPS and LPS + CST injection, Evans Blue (EB, 2% in saline, 4 ml/kg) was injected through the tail vein. Control animals were injected with vehicle (PBS). After 1 h, mice were sacrificed by intraperitoneal injection of pentobarbital and intracardially perfused with PBS. EB quantification was performed as indicated in Additional file 1. Data were represented as μg of dye per mg of brain tissue.

### RNA extraction and determination of gene expression

Following incubation under different conditions, mouse b.End5 and BECs were collected and lysed in TriPure reagent (Roche) for RNA isolation. After RNA isolation, cDNA synthesis from 1 µg RNA was performed using the RevertAid First Strand cDNA Synthesis Kit (ThermoFisher) and random hexamers in a CFX Connect QPCR System (Biorad), under the following conditions: incubation at 25 ºC/5 min, reverse transcription at 42 ºC/60 min, inactivation at 70 ºC/5 min. Gene expression of cortistatin–somatostatin system and inflammation-related genes was determined by a microfluidic-based qPCR dynamic array, as previously described [21]. Conventional qPCR was used to validate differentially expressed genes selected from our transcriptomic studies. Specific primers for mouse transcripts are listed in Additional file 1: Tables S1, S2. Details for each approach are described in Additional file 1.

### Next-generation transcriptome sequencing (RNA-seq)

RNA (200 ng) from primary *Cort*^+*/*+^ and *Cort*^*−/−*^ BECs cultures exposed to NX/NG or OGD-R conditions for 24 h was used to prepare mRNA libraries with Illumina stranded mRNA Prep Ligation kit (Illumina). Three independent biological replicates were performed, with RNA Integrity Number coefficients > 9.9 (Bioanalyzer RNA 6000 Nano-chip, Agilent). Quality and size distribution of mRNA libraries were validated by Bioanalyzer High Sensitivity DNA assay and concentration was measured on the Qubit fluorometer (ThermoFisher). Final libraries were pooled in equimolecular ratios and diluted/denatured as recommended by Illumina NextSeq 500 library preparation guide. The 75 × 2nt paired-end sequencing was conducted on a NextSeq 500 sequencer. The average number of sequencing reads above a quality threshold of Q > 30 was 91.5%. We obtained a mean GC content of 51.3%, and 40,407,022 paired-end reads and > 28,000 transcripts on average. Considering the low levels of cortistatin expression, we have implemented the reanalyzerGSE software [22, 23] for transcriptomic studies. This pipeline was implemented in the RNA-seq analysis to identify differentially expressed genes (DEGs) and to prevent the exclusion of low expressed genes with potential biological relevance (see details in Additional file 1). Both multidimensional scaling (Principal Coordinates Analysis, PCoA) and unsupervised hierarchical clustering of normalized samples were used to assess the divergence and the replicability of the samples included in each one of the independent comparisons (Additional file 1: Fig. S1). DEGs with a False Discovery Rate (FDR) ≤ 0.05 were calculated by comparing: (i) *Cort*^*−/−*^ vs *Cort*^+*/*+^ samples for each condition (NX/NG and OGD-R); (ii) NX/NG vs OGD-R for each genotype (*Cort*^*−/−*^ and *Cort*^+*/*+^). The Fold Change (log_2_FC) was used to evaluate the degree of expression change of each gene. To investigate the potential effects and relevance of DEGs, functional enrichment analyses based on the Gene Ontology (GO) database were performed as indicated in Additional file 1.

### Statistical analysis

Data are expressed as the mean ± SEM. All experiments were randomized and blinded for researchers. The number of independent animals or cell cultures is shown, and statistical analysis was performed using these independent values. Statistical differences comparing two groups were conducted using the unpaired Student's *t* test (for parametric data, and normal distribution) or the non-parametric Mann–Whitney *U* test. For three or more groups with normal distribution and parametric data, one-way ANOVA with appropriate post-hoc tests for multiple comparisons were utilized (with small number of data Bonferroni post-hoc test was preferentially used vs Tukey post-hoc test). Non-parametric data from three or more groups was analysed by Kruskal–Wallis test and Dunn’s post-hoc test. When standard deviations were assumed as different, Brown–Forsythe and Welch ANOVA test were applied with Dunnett T3 post-hoc test. Spearman’s rho non-parametric test was used for correlation studies. All analyses were performed using GraphPad Prism v8.3.0 software. We considered *P* values < 0.05 (two-tailed) as significant.

### Data availability

RNA-seq data are available in the GEO repository under accession number GSE207405, https://www.ncbi.nlm.nih.gov/geo/query/acc.cgi?acc=GSE207405.

## Results

### Brain endothelium expresses the components of the cortistatin pathway

ECs from peripheral murine and human vessels express the neuropeptides somatostatin, cortistatin, and ghrelin, and their corresponding receptors [24, 25]. However, the expression of these neuropeptides and their receptors in brain ECs is either absent or poorly documented [24, 26]. Thus, using methodologies with different sensitivity, we first investigated the relative expression of the components related to the cortistatin–somatostatin system in b.End5 murine brain ECs. A variable expression level for each SSTR was found under physiological conditions (Additional file 1: Fig. S2a, b). We observed that *Sstr4,* *Sstr1* and *Sstr2* seem to be preferentially expressed, while *Sstr3*, *Sstr5* *and* *Ghsr* levels were low or undetectable. Low levels of cortistatin and somatostatin, were also detected by both Fluidigm and conventional qPCR techniques. Interestingly, we found a preferential expression for endogenous *Cort* vs *Sst* in the brain endothelial cells (Additional file 1: Fig. S2a, b).

### Cortistatin regulates the permeability and integrity of the brain vascular endothelium after injury

**Fig. 1:**
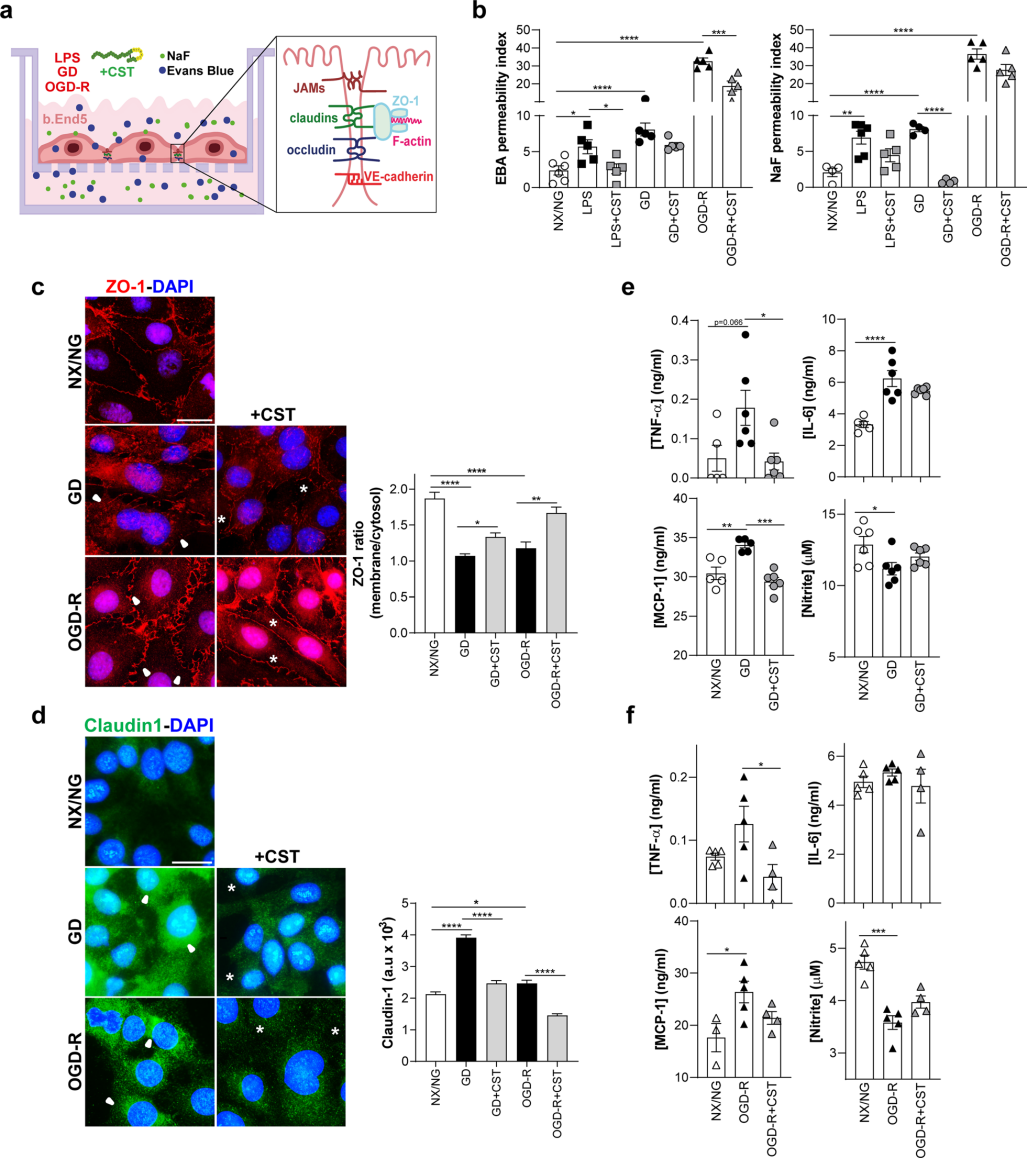
Cortistatin regulates the integrity of b.End5 cells exposed to different insults. **a** Schematic representation of the experimental design. Murine b.End5 cells were exposed to lipopolysaccharide (LPS), glucose deprivation (GD) for 24 h, or to 4 h of oxygen–glucose deprivation followed by 20 h of reoxygenation (OGD-R). Cortistatin (CST, 100 nM) was applied simultaneously to the insult (LPS + CST; GD + CST) or during recovery (OGD-R + CST). Cells incubated in normoxia/normoglycemia (NX/NG) were used as a reference. **b–d** Endothelial integrity was assessed by evaluating permeability to Evans Blue-Albumin (EBA) and Sodium Fluorescein (NaF) (**b**), and tight-junctions assembly (**c, d**). **b** Permeability is represented as the index (%) of the tracer permeability vs an empty-coated insert. *N* = 4–6 cultures/group. **c, d** Representative immunofluorescence images of the cellular distribution of ZO-1 (**c**, red) and claudin-1 (**d**, green) in b.End5 cells after GD or OGD-R in the absence or presence of cortistatin. Delocalization of ZO-1 was evaluated through the ratio of ZO-1 staining intensity in the membrane vs the cytosol (**c**, arrowheads indicate gaps in ZO-1 membrane location). After cortistatin addition, ZO-1 junctional rearrangement was observed in the membrane (asterisks). Claudin-1 intracellular overexpression was quantified by fluorescence intensity (**d**). Cytosolic expression of claudin-1 was augmented in GD/OGD-R (arrowheads). Cortistatin drastically decreased claudin-1 intracellular expression (asterisks). 25–50 selected ROIs in 4 independent fields were analysed (expressed as arbitrary units, a.u). *N* = 6 cultures/group. Scale bar: 20 µm. **e, f** Levels of inflammatory cytokines TNF-α, IL-6, chemokine MCP-1 (all in ng/ml) and nitrite (µM) were determined in culture supernatants after GD (**e**) or OGD-R (**f**) with or without cortistatin addition. *N* = 4–6 cultures/group. Data are the mean ± SEM with dots representing individual values of the independent cultures. *p ≤ 0.05, **p ≤ 0.01, ***p ≤ 0.001, ****p ≤ 0.0001

BBB breakdown is a hallmark in most neurodegenerative/neuroinflammatory disorders and injured brain ECs are enough to disrupt the BBB. In this sense, previous results showed the critical role of cortistatin in the regulation of the peripheral vascular endothelium [13–15]. Therefore, we evaluated the possible influence of this neuropeptide on the integrity of the brain endothelium. First, we characterized the effect of cortistatin in b.End5 cells exposed to different insults mimicking brain injury-like conditions (Fig. 1a).

Specifically, we observed increased endothelial cell permeability after incubation with bacterial LPS, glucose deprivation (GD), or oxygen–glucose deprivation and reoxygenation (OGD-R) (Fig. 1b). However, the addition of exogenous cortistatin to b.End5 cultures significantly reversed this increased permeability (Fig. 1b). The lack of endothelial integrity was accompanied by a delocalization and disruption of the TJ assembly (Fig. 1c, d). GD and OGD-R caused the disruption of ZO-1 (Zonula occludens-1) uniform pattern in the membrane (Fig. 1c), as well as the increase of claudin-1 intracellular expression (correlated with BBB disruption, as shown by [27]) (Fig. 1d).

Cortistatin restored ZO-1 integrity in the peripheral cell membrane (Fig. 1c) and significantly reduced claudin-1 cytosolic accumulation under both conditions (Fig. 1d). Moreover, injured ECs developed an altered inflammatory response, characterized by an augmented production of the proinflammatory factors IL-6 and MCP-1, and reduced levels of nitrite, quantified as a major metabolite of nitric oxide, all linked to BBB leakage [16] (Fig. 1e, f). Cortistatin treatment exerted an immunomodulatory effect on brain endothelium activation by significantly downregulating TNF-α and MCP-1, without affecting nitrite levels (Fig. 1e, f). Moreover, cortistatin addition seems to modulate to basal levels the OGD-R-derived dysregulated expression of other immune mediators, inflammasome components and endothelial-derived agents being only significant for *Il6ra* (Additional file 1: Fig. S3a–c). Although some biological trends can be observed, exposure to OGD-R and/or treatment with cortistatin did not significantly affect the expression levels of *Cort*, *Sst* or their receptors (Additional file 1: Fig. S2c). Notably, during injury conditions, the secreted levels of endogenous cortistatin were reduced significantly (Additional file 1: Fig. S2d).

**Fig. 2:**
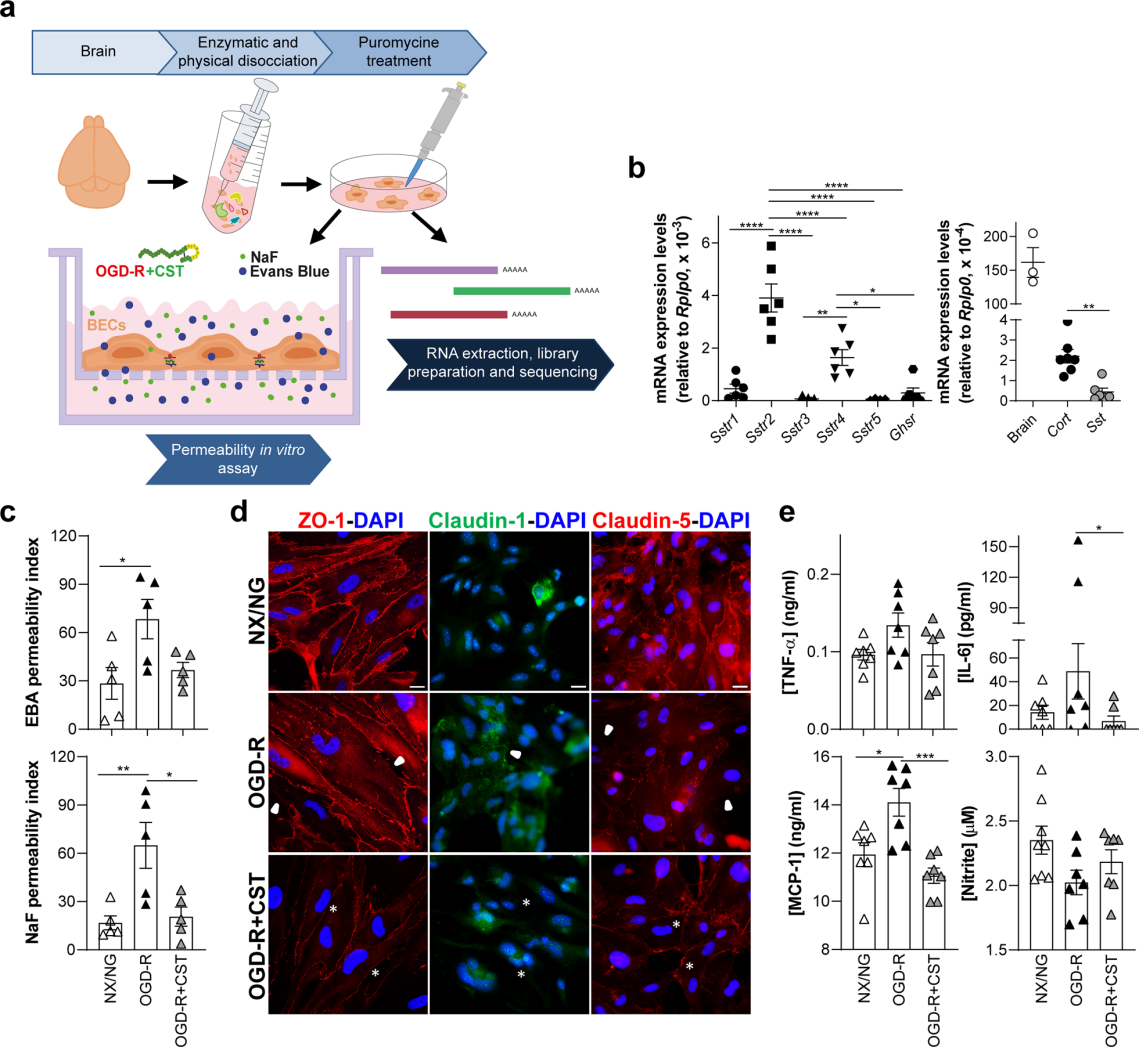
Cortistatin reverses murine brain endothelium disruption after ischemic-like conditions. **a** Graphical illustration of the experimental design. Primary brain endothelial cells (BECs) were isolated from 8-week-old *Cort*^+*/*+^ mice and enriched after 60 h puromycin treatment. One week later, cells were plated in collagen/fibronectin-coated transwells as described before. BECs were exposed to OGD-R with or without cortistatin (CST, 100 nM) and gene profile expression analysis or permeability assays were performed. **b** mRNA expression levels of cortistatin system receptors (left, *Sstr* and *Ghsr1a*) and ligands (right, somatostatin, *Sst* and cortistatin, *Cort*) in the brain endothelium under NX/NG. Data represent the mean of mRNA expression levels quantified by real-time qPCR analyses and normalized to *Rplp0*. *N* = 5–7 cultures/group. Mouse brain (*n* = 3) was used as an internal reference for endogenous *Cort* expression. **c** Endothelium barrier functionality was assessed by evaluating permeability to EBA and NaF and tight-junctions integrity. Index (%) of the tracer permeability vs an empty-coated insert. *N* = 5 cultures/group. **d** Representative immunofluorescence images of the cellular distribution of ZO-1 (red), claudin-1 (green) and claudin-5 (red) in BECs after OGD-R in the absence or presence of cortistatin. Arrowheads indicate ZO-1 and claudin-5 disruption throughout the cell membrane and augmented cytosolic expression of claudin-1. After cortistatin treatment, asterisks point out the recovery of ZO-1 and claudin-5 continuous pattern in the membrane and the reduced claudin-1 intracellular location. Scale bar: 20 µm. **e** Levels of immune factors were determined in culture supernatants after OGD-R in the presence or absence of cortistatin. N = 7 cultures/group. Data are the mean ± SEM with dots representing individual values of independent cultures. Cells for each culture derived from 4 pooled brains. *p ≤ 0.05, **p ≤ 0.01, ***p ≤ 0.001, ****p ≤ 0.0001

Despite the fact that immortalized mouse brain endothelioma cell lines have been widely used, primary murine brain endothelial cells (BECs) have been shown to better retain several phenotypic properties of the BBB in vitro, due to their specialization with complex TJs [28]. Thus, we examined the role of cortistatin in murine BECs isolated from the adult CNS microvasculature (Fig. 2a). As described above for the cell line, cortistatin–somatostatin system was also expressed by primary BECs under homeostatic conditions. Although we found some variability for the expression levels of the receptors and neuropeptides according to the different methodologies, low levels of *Sstr3* and *Ghsr1a* were observed, while *Sstr2* and *Sstr4* significantly displayed the highest expression (Fig. 2b; Additional file 1: Fig. S4). Of note, endogenous *Cort* expression was higher compared to *Sst*. Next, to further support our previous results with b.End5, BECs were incubated under OGD-R as the preferred insult to mimic the model of ischemic–reperfusion injury to study BBB disruption. ECs significantly increased permeability, showing a compromised TJ architecture and an exacerbated immune response (Fig. 2c, d). Treatment with exogenous cortistatin reversed brain endothelial cell dysfunction back to the homeostatic state, decreasing permeability, preserving functional proteins for the barrier formation (Fig. 2d) and downregulating the production of immune mediators, such as MCP-1 and IL-6 (Fig. 2e). Importantly, secretion of cortistatin was reduced under OGD-R conditions (Additional file 1: Fig. S6c).

**Fig. 3:**
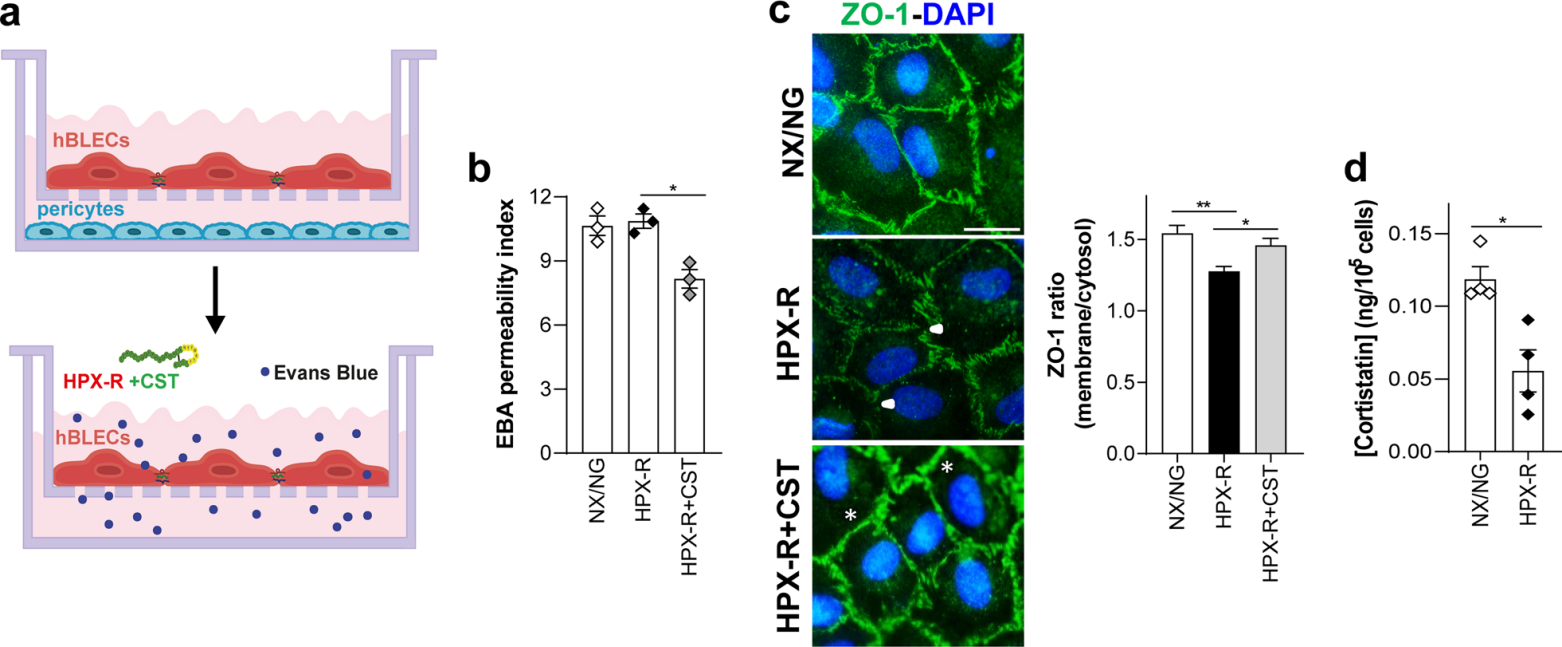
Role of cortistatin in the human brain endothelium. **a** Human brain-like endothelial cells (hBLECs) were obtained after 1 week of co-culture of CD34+-derived endothelial cells with pericytes. hBLECs were incubated 4 h under hypoxia followed by 20 h of reoxygenation (HPX-R) in the absence or presence of cortistatin (HPX-R + CST, 100 nM). **b** Endothelium integrity was evaluated and represented as the index (%) of EBA permeability vs an empty-coated insert. *N* = 3 cultures/group. Data are the mean ± SEM with dots representing individual values of independent cultures. **c** *Left*, expression of ZO-1 (green) was evaluated by immunofluorescence. Arrowheads indicate reduced and disrupted expression of ZO-1 after HPX-R. Cortistatin treatment protected the membrane from breakdown and recovered the tight pattern of intercellular ZO-1 (asterisks). *Right*, delocalization of ZO-1 was evaluated through the ratio of ZO-1 staining intensity in the membrane vs the cytosol. 25–50 selected ROIs in 4 independent fields were analysed. *N* = 6 cultures/group. **d** Cortistatin protein levels were quantified in hBLECs supernatants 20 h after exposure to NX/NG and HPX-R, as described. Results are normalized in ng protein/10^5^ cells. *N* = 4 cultures/group. *p ≤ 0.05, **p ≤ 0.01. Scale bar: 20 µm

In addition, we also evaluated the potential role of cortistatin in human-derived brain ECs under ischemic–reperfusion injury mimicked by hypoxia– and oxygen–glucose deprivation assays combined with reoxygenation (Fig. 3; Additional file 1: Fig. S5).

Although no changes were detected in the endothelial barrier permeability between NX/NG and HPX-R conditions (Fig. 3b), probably due to the variability of serum-derived effects [29, 30], only the injured endothelial cells showed a disruptive presence of ZO-1 (Fig. 3c). Incubation with cortistatin reduced permeability in hypoxic cells and recovered intact ZO-1 presence in the cell membrane (Fig. 3b, c). As expected, OGD-R did induce an increase in barrier permeability of hBLECs. This increase was associated with a reduced expression of ZO-1 and an increase in ZO-1 disorganization, as well as claudin-5 membrane depletion (Additional file 1: Fig. S5a, b). Paradoxically, the addition of cortistatin significantly recovered the junctional expression and architecture of ZO-1 and claudin-5, although no changes were observed in the permeability of OGD-R-injured cells (Additional file 1: Fig. S5a, b). While discontinuous junctions are typically indicative of decreased barrier function and increased permeability, recent works have demonstrated that global permeability measures (as the ones used for transwell experiments) may not correlate with the junction phenotype (i.e., permeability measure remains the same with continuous and discontinuous arrangement of TJs) [31]. Interestingly, as we observed in the murine endothelial cells, healthy hBLECs predominantly expressed cortistatin, while somatostatin levels were almost undetectable (Additional file 1: Fig. S5c). Of note, production of cortistatin was regulated upon ischemic and reoxygenation damage, as the endogenous secretion of the neuropeptide was significantly reduced (Fig. 3d; Additional file 1: Fig. S5d).

Altogether, these data suggest that cortistatin-system (and not somatostatin) could play a crucial role in regulating brain endothelium integrity at different levels after several brain injury-like conditions.

### Deficiency in cortistatin exacerbates a dysfunctional brain endothelium response

**Fig. 4:**
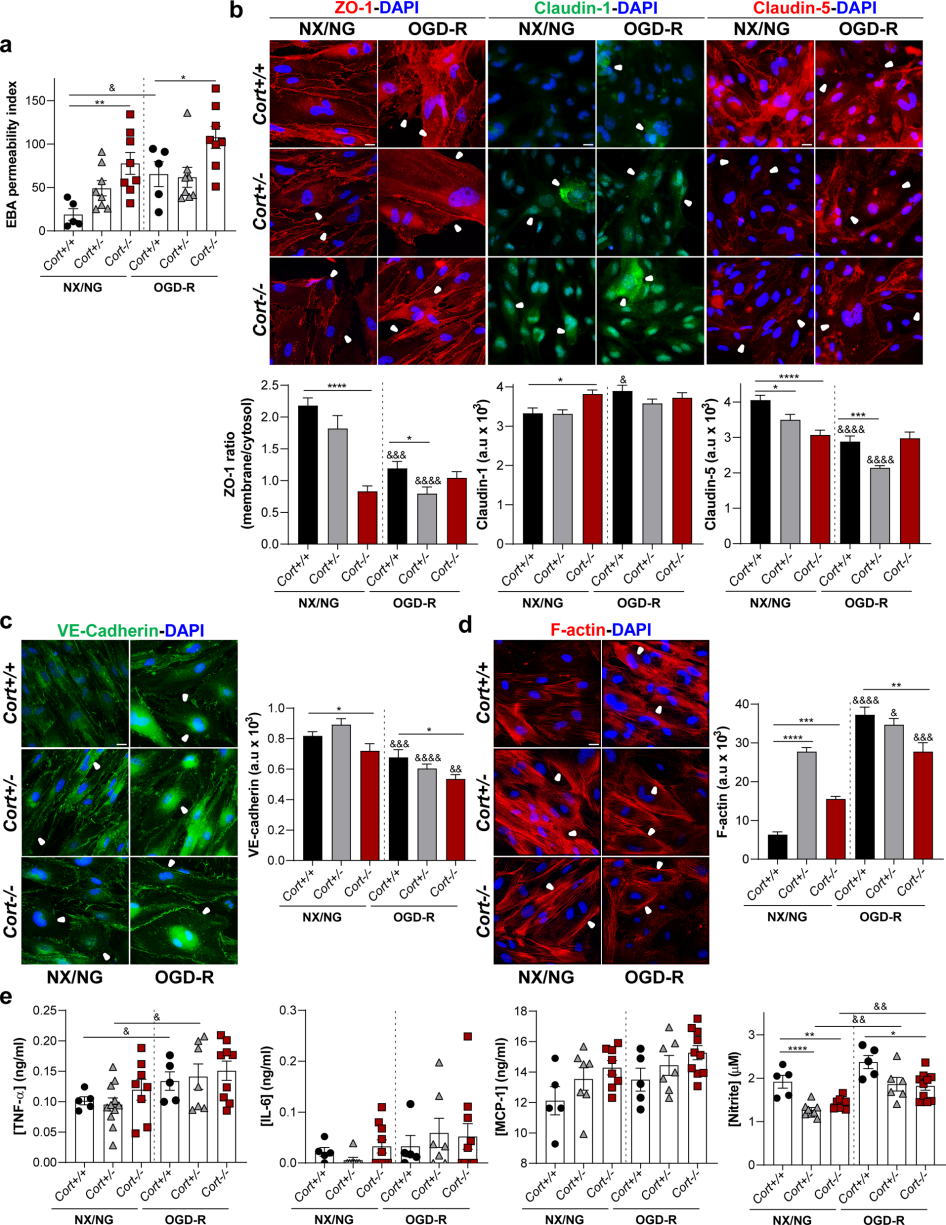
Cortistatin-deficient brain endothelial cells (BECs) show increased leakage and exacerbated immune activation. **a** BECs were isolated from wild type (*Cort*^+*/*+^) and cortistatin-deficient (partial: *Cort*^+/−^; complete: *Cort*^*−/−*^) mice and exposed to 4 h oxygen–glucose deprivation followed by 20 h of reoxygenation (OGD-R). Barrier integrity was evaluated and represented as the index (%) of EBA permeability vs an empty-coated insert. *N* = 5–8 cultures/group. **b–d** Representative immunofluorescence images of cellular junctions distribution in BECs incubated in basal conditions (NX/NG) and after OGD-R. **b** Immunofluorescence of tight-junctions ZO-1 and claudin-5 (both in red, arrowheads point out membrane disruption) and claudin-1 (green, arrowheads indicate augmented cytosolic expression). Delocalization of ZO-1 was evaluated through the ratio of ZO-1 staining intensity in the membrane vs the cytosol. Claudin-1 and claudin-5 cellular expression was quantified by fluorescence intensity as described below. **c, d** Adherens-junction protein VE-cadherin (**c**, green, arrowheads show discontinuous expression) and stress fibers (**d**, red rhodamine–phalloidin F-actin labelling, arrowheads reveal cytosolic reorganization of F-actin thick bundles) were analysed by quantifying fluorescence intensity. 25–50 selected ROIs in 4 independent fields were analysed (expressed as arbitrary units, a.u). *N* = 6 cultures/group. Scale bar: 20 µm. **e** Levels of inflammatory factors were determined in culture supernatants after OGD-R. *N* = 5–10 cultures/group. Data are the mean ± SEM with dots representing individual values of independent cultures. Cells for each culture were derived from 4 pooled brains. *vs *Cort*^+*/*+^ either in NX/NG and OGD-R; ^&^vs corresponding genotype (*Cort*^+*/*+^, *Cort*^+/−^, *Cort*^*−/−*^) in NX/NG. *^/&^*p* ≤ 0.05, **^/&&^*p* ≤ 0.01, ***^/&&&^*p* ≤ 0.001, ****^/&&&&^*p* ≤ 0.0001

Previous studies demonstrated the regulatory properties of cortistatin in the peripheral vascular system, including exacerbated responses to vascular lesions in cortistatin-deficient mice [13–15]. In addition, our findings demonstrate that cortistatin modulates the dynamics of the brain endothelium and that both murine and human brain ECs respond to pathological conditions by modifying the expression of cortistatin (Figs. 1, 2, 3). To further investigate whether endogenous cortistatin plays a role in the control of brain endothelium functionality, we characterized the phenotype and dynamics of BECs isolated from the brain microvasculature of wild-type (*Cort*^+*/*+^) and cortistatin-deficient mice (*Cort*^+/−^ and *Cort*^*−/−*^) under basal (NX/NG) and pathological (OGD-R) environments (Fig. 2a). Compared to wild-type BECs, cortistatin-deficient cultures showed a significant increase in endothelial permeability, not only after the insult but also when receiving a continuous supply of oxygen and nutrients (Fig. 4a; Additional file 1: Fig. S6a). Our data showed that the enhanced permeability observed with partial/complete absence of cortistatin was correlated with a greater delocalization, disruption and dysfunctional assembly of the TJ/AJ in both ischemic and normal environments (Fig. 4b–d). Specifically, by determining the ratio between the location of ZO-1 in the membrane vs the cytosol, we demonstrated that, under both conditions (NX/NG and OGD-R), a dramatic disintegration and redistribution of this protein occurred in the absence of cortistatin (Fig. 4b). Moreover, claudin-1 was overexpressed in BECs cultures without cortistatin (Fig. 4b). Similar to ZO-1, the breakdown of claudin-5 and the AJ VE-cadherin showed junctional discontinuity and a significant reduction in the expression in cortistatin-deficient BECs compared to control cells (Fig. 4b, c).

In addition, to address the important role of the cytoskeleton-junctional proteins assembly in the modulation of BBB leakage, we performed a rhodamine–phalloidin labelling (Fig. 4d). While F-actin was distributed in random fibers throughout the cytosol in NX/NG wild-type BECs, it was drastically reorganized into thick stress bundles in all genotypes after injury. Interestingly, the absence of cortistatin also promoted a significant accumulation of stress fibers in physiological conditions (Fig. 4d). Notably, all these changes affecting TJ/AJ architecture were mostly appreciated in BECs with complete lack of the neuropeptide. The hyperpermeability observed in BECs without cortistatin was also accompanied by an altered inflammatory phenotype (Fig. 4e). Notably, we found a downregulated production of nitrite not only after the insult but also under normal conditions in the absence of cortistatin (Fig. 4e).

Next, we checked the regulation of the components of the cortistatin system in the absence of the neuropeptide (Additional file 1: Fig. S6b). Although the expression levels of the receptors showed no changes when comparing BECs isolated from cortistatin-deficient mice to the expression levels in wild-type cells under NX/NG, we found a partial non-significant decrease in the physiological levels of *Sstr1,* *Sstr5* and *Sst* that was still reduced in the OGD-R environment. Importantly, only *Sstr4* was significantly and oppositely modulated after injury in wild type and cells lacking cortistatin (Additional file 1: Fig. S6b). Interestingly, endogenous levels of cortistatin in healthy heterozygous BECs were similar to those secreted by wild-type cells under hypoxic–reoxygenation conditions (Additional file 1: Fig. S6c).

Taken together, these findings indicate that the brain ECs from mice lacking cortistatin exhibit a leaky and inflammatory-like endothelium already under physiological conditions, being exacerbated under damage. Our results suggest that the system conformed by endogenous cortistatin (and probably, by *Sstr4/Sstr2/Sstr5*) might be crucial in the regulation of the brain endothelium dynamics during physiology and pathology, which could be determinant for BBB integrity.

### Cortistatin-deficient brain endothelium shows a dysfunctional phenotype with dysregulated physiological pathways

To further investigate the molecular profile that could be linked to the cerebral vascular endothelium behaviour in the absence of cortistatin, we compared the transcriptomes of *Cort*^+/+^ and *Cort*^−/−^ BECs under NX/NG or OGD-R obtained by next-generation RNA-seq. First, we confirmed that our samples showed strong enrichment of BECs-specific markers (such as *Cldn5,* *Pecam1/*CD31 or *Tjp1/*ZO-1), while cells expressing markers of pericytes (*Cspg4,* *Kcnj8,* *Vtn*), astrocytes (*Gfap,* *Aqp4,* *Gli1*), neurons (*Nefl,* *Gabra1,* *Reln*), oligodendrocytes (*Olig1,* *Mog,* *Mbp*), or microglia (*Ptprc,* *Itgam,* *Tnf*) were barely detectable (Additional file 1: Fig. S7a).

**Fig. 5:**
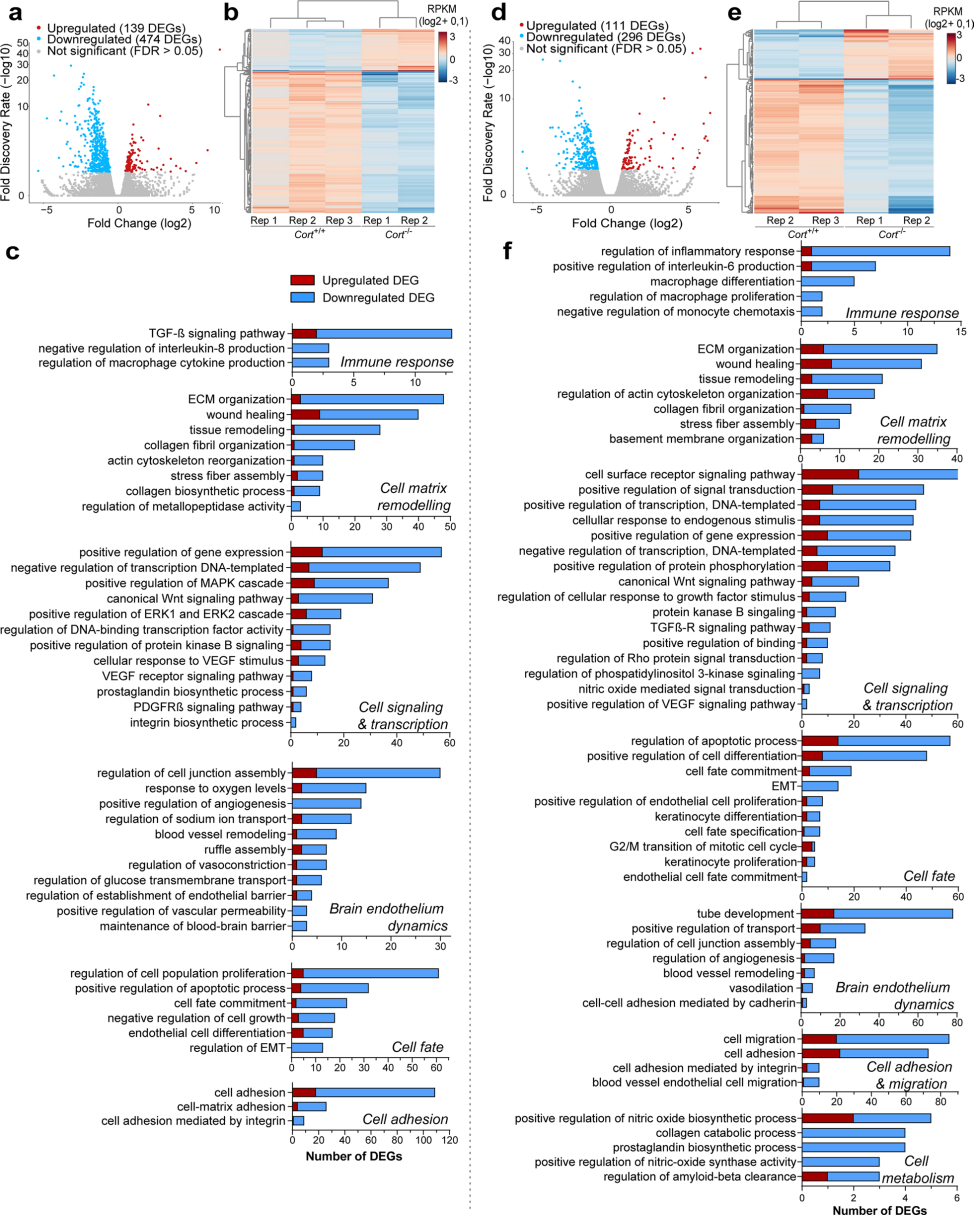
Cortistatin-deficient brain endothelium shows deregulated gene pathways. Comparison of gene expression profiles from BECs isolated from *Cort*^+*/*+^ vs *Cort*^*−/−*^ mice cultured as described in Fig. 2a. Each biological replicate (Rep) was pooled from 3 mice per genotype. **a, d** Volcano plots illustrate differentially expressed genes (DEGs) from *Cort*^*−/−*^ vs *Cort*^+*/*+^ BECs incubated under NX/NG for 24 h (**a**) or under 4 h oxygen–glucose deprivation followed by 20 h reoxygenation (OGD-R) (**d**). Each dot represents one gene. Grey dots represent not significantly altered genes. The number of enriched (up, red) and decreased (down, blue) genes (with false discovery rate, FDR, *p* < 0.05) for cortistatin-deficient BECs is shown in the legend. Full description of DEGs is in Additional file 2: Tables S3 and S4. **b, e** Heatmaps and unsupervised hierarchical clustering of 613 DEGs (**b**) and 407 DEGs (**e**) in *Cort*^*−/−*^ vs *Cort*^+*/*+^ BECs exposed to NX/NG and OGD-R, respectively. The expression values are represented in shades of red and blue, indicating expression above and below the median value across all samples. **c, f** Gene ontology (GO) terms for biological processes significantly overrepresented in *Cort*^*−/−*^ vs *Cort*^+*/*+^ BECs incubated under NX/NG (**c**) or OGD-R (**f**). Data were grouped into similar gene sets and manually annotated into several networks, such as immune response, cellular matrix and remodelling, cell signalling and transcription, cell fate, brain endothelium dynamics, cell adhesion and migration, and cell metabolism. Red and blue bar-segments correspond to the numbers of upregulated and downregulated DEGs, respectively. All DEGs for each GO term are listed in Additional file 2: Tables S5 and S6. TGF-β, transforming growth factor beta; ECM, extracellular matrix; MAPK, mitogen-activated protein kinase; ERK, extracellular-signal-regulated kinase; VEGF, vascular endothelial growth factor; PDGFRβ, platelet-derived growth factor receptor beta; EMT, epithelial to mesenchymal transition

In brain endothelium from cortistatin-deficient mice, we identified 613 genes (139 upregulated and 474 downregulated) with significant differential expression (FDR < 0.05) compared to wild type in an NX/NG environment (Fig. 5a; Additional file 2: Table S3).

On the other hand, we observed that 407 genes (111 upregulated and 296 downregulated) were differentially expressed between *Cort*^−/−^ and *Cort*^+/+^ BECs after OGD-R exposure (Fig. 5d; Additional file 2: Table S4). The unsupervised hierarchical clustering analysis revealed two distinct groups with minimal overlap under both experimental conditions (Fig. 5b, e). Next, we validated by qPCR the expression of key genes up and downregulated, showing a high degree of correlation with the RNA-seq quantification (Additional file 1: Fig. S7b, c). Notably, in cortistatin-deficient BECs, we observed that 226 DEGs (more than half out of the total) were shared between NX/NG and OGD-R when comparing *Cort*^+/+^ vs *Cort*^−/−^ BECs. Through GO terms overrepresentation analysis corresponding to biological processes (BP), molecular functions (MF) and cellular components (CC), we showed that most DEGs were associated with downregulated functional networks (Fig. 5c, f; Additional file 1: Fig. S8, Additional file 2: Tables S5, S6).

**Fig. 6:**
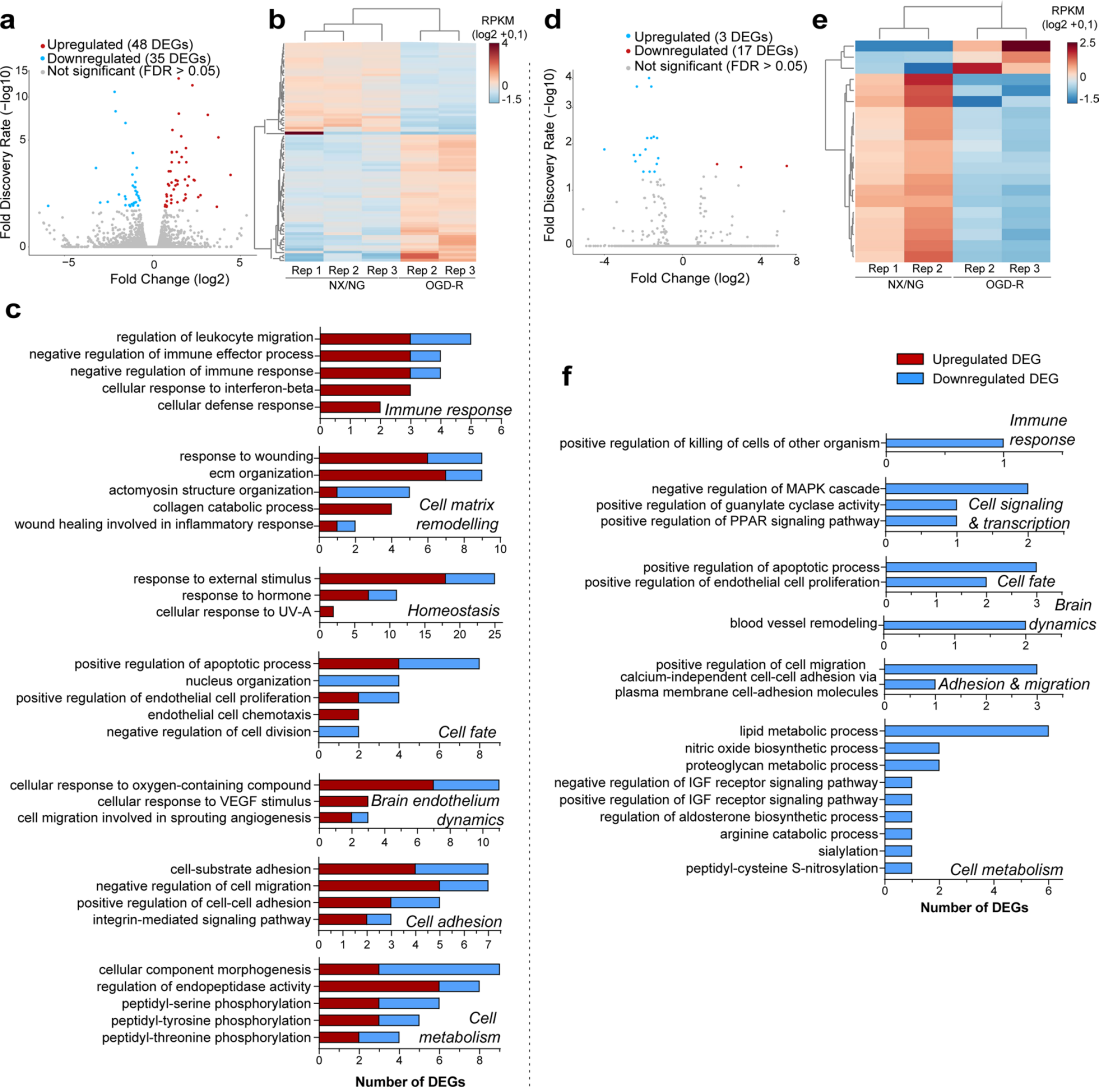
Gene networks regulating brain endothelium dynamics are affected by the lack of cortistatin. Comparison of gene expression profiles in the dynamics from the basal (NX/NG) to ischemic and repairing (OGD-R) states in *Cort*^+*/*+^ and *Cort*^*−/−*^ BECs. Each biological replicate (Rep) was pooled from 3 mice per genotype. **a**, **d** Volcano plots illustrating DEGs between BECs incubated under NX/NG for 24 h and BECs exposed to 4 h oxygen–glucose deprivation and 20 h reoxygenation (OGD-R) from *Cort*^+/+^ (**a**) or *Cort*^*−/−*^ (**d**). Each dot represents one gene. Grey dots represent not significantly altered genes. The number of enriched (up, red) and decreased (down, blue) genes (FDR, *p* < 0.05) for OGD-R-derived BECs is shown in the legend. Full description of DEGs is in Additional file 2: Tables S7 and S8. **b, e** Heatmaps and unsupervised hierarchical clustering of 83 DEGs (**b**) and 20 DEGs (**e**) in BECs exposure to NX/NG vs OGD-R in *Cort*^+/+^ and *Cort*^−/−^, respectively. Expression values are represented in shades of red and blue, indicating expression above and below the median value across all samples. **c, f** GO terms for biological processes significantly overrepresented in BECs incubated under NX/NG vs OGD-R from *Cort*^+/+^ (**c**) and *Cort*^*−/−*^ (**f**) mice. Data were grouped into similar gene sets and manually annotated into several networks, such as immune response, cellular matrix and remodelling, homeostasis, cell signalling and transcription, cell fate, brain endothelium dynamics, cell adhesion and migration, and cell metabolism. Red and blue bar-segments correspond to the numbers of upregulated and downregulated DEGs, respectively. All DEGs for each GO term are listed in Additional file 2: Tables S9 and S10. *VEGF* vascular endothelial growth factor, *ECM* extracellular matrix, *MAPK* mitogen-activated protein kinase, *PPAR* peroxisome proliferator-activated receptor

In fact, these gene clusters, i.e.*,* cell matrix remodelling, brain endothelium dynamics, immune response, regulation of gene expression (through MAPK, Wnt, VEGF, and Erk1/2 pathways), growth factors activity (BMP, IGF-II, VEGF and TGF-β, among others), endothelial cell fate commitment, and endothelium-dependent cell adhesion and migration, were similarly affected by both conditions (Fig. 5c, f; Additional file 1: Fig. S8, Additional file 2: Tables S5, S6). These results indicate that the lack of cortistatin seems to induce downregulated pathways affecting both normal brain endothelium processes and injured-endothelium responses. To investigate the possible dysfunctional phenotype of brain endothelium from mice with cortistatin deficiency under physiological conditions, we compared the transcriptional programs associated with the progression from a healthy (NX/NG) to an injured state followed by reoxygenation (OGD-R), for both *Cort*^+/+^ and *Cort*^−/−^ BECs. Our results revealed that 83 genes (48 upregulated and 35 downregulated) were differentially expressed in wild-type cells when driving the transition from NX/NG to OGD-R (Fig. 6a, b; Additional file 2: Table S7).

Noteworthy, resulting GO terms revealed that upregulated DEGs found in *Cort*^+/+^ BECs under OGD-R were positively associated with gene networks modulating endothelial injury responses (Fig. 6c; Additional file 1: Fig. S9a, Additional file 2: Table S9). Remarkably, these pathways were not only related to the deleterious response to damage, but also to several processes aimed at a later balanced repair response, such as the immune regulation of leukocyte migration, the response to immune mediators, the remodelling of the extracellular matrix and cytoskeleton, the regulation of angiogenesis and wound healing processes, and the activation of protective stress responses (Fig. 6c; Additional file 1: Fig. S9a). Surprisingly, in the dynamics of *Cort*^−/−^ BECs from NX/NG to OGD-R, only 20 differentially expressed genes (3 upregulated and 17 downregulated) were identified in the transition from a healthy state to an ischemic and reperfusion context (Fig. 6d, e; Additional file 2: Table S8). In this case, the genes were predominantly involved in downregulated networks associated with the response to damage (Fig. 6f; Additional file 1: Fig. S9b, Additional file 2: Table S10).

**Fig. 7:**
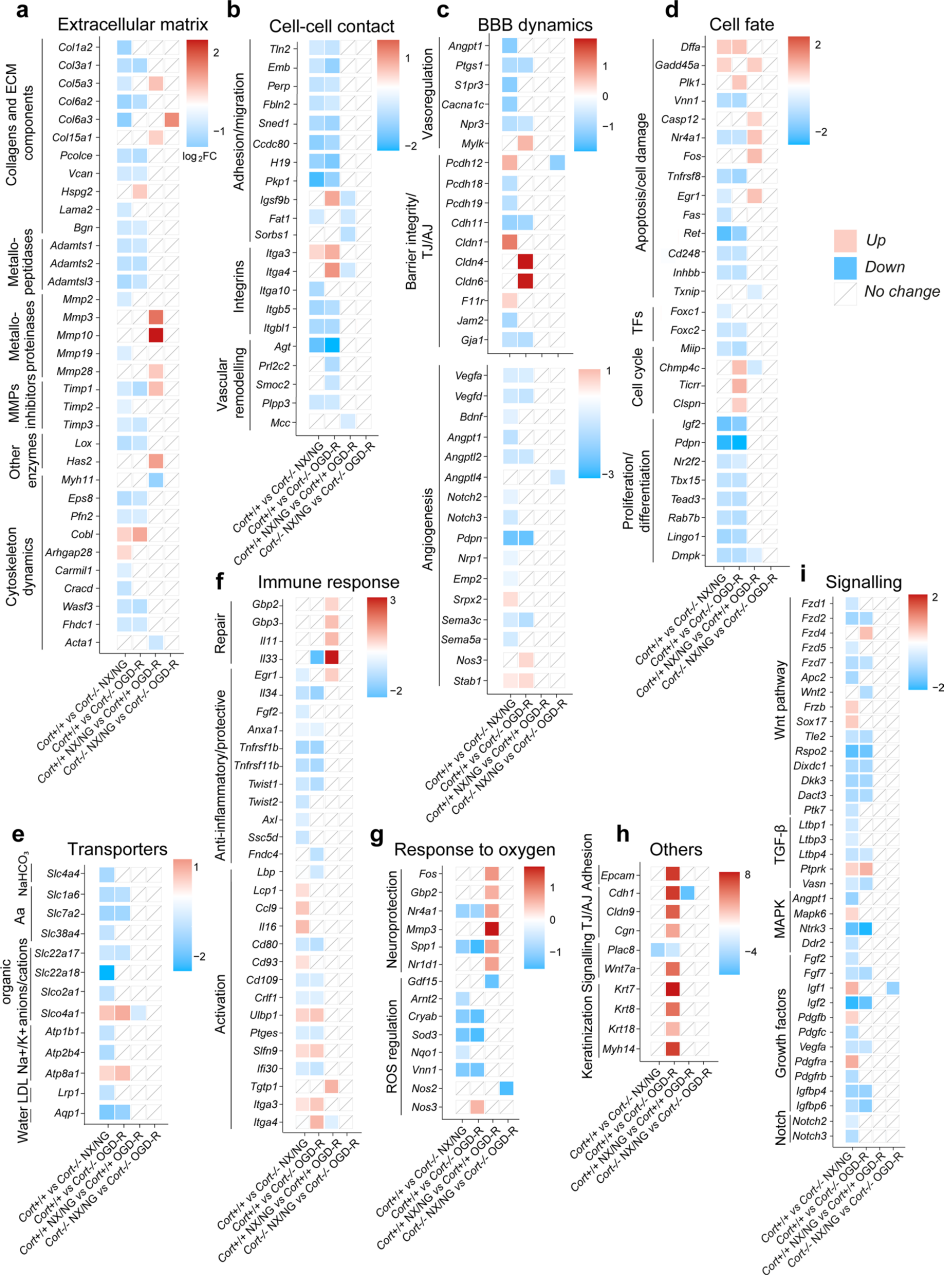
Transcriptional alterations in cortistatin-deficient brain endothelium. Gene sets for *Cort*^+*/*+^ and *Cort*^*−/−*^ BECs displayed in Figs. 5 and 6 were manually annotated into gene modules relevant for key BBB features and functions: **a** extracellular matrix components; **b** cell–cell contact mediators; **c** BBB dynamics; **d** cell fate agents; **e** endothelial transporters; **f** immune response; **g** response to oxygen; **h** others, (genes with the highest fold change from different categories); and **i** signalling pathways. Differential expression patterns of selected DEGs modulated by the lack of cortistatin are represented in each module based on criteria defined in the legend box. Genes that do not have significant expression changes are displayed by a white box. Relative fold change expression (log_2_FC) in each experimental group is shown by shades of red (upregulated) and blue (downregulated). *MMPs* matrix metalloproteinases, *TFs* transcription factors, *Aa* amino acids, *LDL* low density lipoprotein, *MAPKs* mitogen-activated protein kinases

Next, we performed a more detailed examination of the DEGs conforming these pathways based on their log_2_ fold change and their biological relevance to brain endothelial function (Additional file 2: Table S11). Analysis of the extracellular matrix (ECM) remodelling cluster (Fig. 7a), revealed that most of the genes encoding collagens and other ECM components, metalloproteinases inhibitors, metallopeptidases as well as genes regulating actin cytoskeleton dynamics, among others, were downregulated in the basal (NX/NG) and injured (OGD-R) brain endothelium when cortistatin was absent.

Notably, additional genes related to ECM remodelling, i.e*.*, *Col1a2,* *Col6a3,* *Lama2,* *Mmp2,* *Mmp19,* *Timp2*, *Carmil1,* and *Cracd,* and to cytoskeleton disassembly, *Arhgap28* and *Cobl* were downregulated and upregulated, respectively, in cortistatin-deficient BECs only under NX/NG (Fig. 7a). On the contrary, the matrix components *Col5a3,* *Col15a1* and *Has2* (hyaluronic acid), the metalloproteinases *Mmp3,* *Mmp10* or *Mmp28* and the metalloproteinase inhibitor *Timp1* were upregulated in *Cort*^+*/*+^ cells under OGD-R conditions, following the canonical response to damage and recovery (Fig. 7a). However, in *Cort*^*−/−*^ cells during the progression from NX/NG to OGD-R, none of these or related genes demonstrated differential expression. In addition, several genes involved in cell adhesion/migration, as well as some integrins, were downregulated during NX/NG and OGD-R in cortistatin-deficient cells compared to wild-type endothelium (Fig. 7b). Of note, *Itga3* and *Itga4*, involved in the chemoattraction and activation of immune cells, were upregulated in the absence of cortistatin (Fig. 7b).

Furthermore, we observed that genes linked to BBB dynamics were affected. We found that many master regulators of angiogenesis vascular remodelling pathways, as well as genes involved in vasoregulation, were already downregulated in *Cort*^−/−^ BECs under NX/NG and some also reduced after OGD-R (Fig. 7b, c). Moreover, some genes related to the integrity of the BBB were downregulated in *Cort*^−/−^ BECs under NX/NG, OGD-R or both conditions (Fig. 7c). On the contrary, only a few pro-angiogenic genes such as *Srpx2,* *Stab1* or Notch-dependent *Pcdh12* were upregulated. Interestingly, genes linked to BBB breakdown and TJ destruction [27, 32, 33] were upregulated during OGD-R (*Cldn4,* *Cldn6*, *Cldn9*, *Cdh1*, *Mylk*, and *Myh14*) or even in the physiological state of the cortistatin-deficient brain endothelium (*Cldn1* and *F11r*) (Fig. 7c, h).

In addition, we observed that the absence of cortistatin induced a marked downregulation of factors that affect endothelial proliferation and differentiation, as well as those associated with apoptosis and the promotion of cell survival (Fig. 7d). Furthermore, genes related to DNA damage were upregulated. Interestingly, some genes linked to cell cycle such as *Chmp4c* and *Ticrr* were also upregulated (Fig. 7d).

Some of these genes, required to the response to damage (*Casp12,* *Egr1,* *Fos* and *Nr4a1*) [34], were upregulated in the dynamic transition from NX/NG to OGD-R for *Cort*^+*/*+^ BECs, whereas none of them showed changes in the same process in endothelium lacking cortistatin (Fig. 7d, g).

Moreover, we detected that the network involving BBB transporters, crucial for metabolic and ionic endothelial homeostasis, was downregulated in the absence of cortistatin in both, control and injury conditions (Fig. 7e). On the contrary, *Slco4a1* and *Atp8a1*, detrimental to brain connectivity when elevated [35, 36], were upregulated in *Cort*^−/−^ BECs in basal and injured states (Fig. 7e).

Regarding genes involved in immune pathways, our results revealed that factors linked to an anti-inflammatory, immunoregulatory and protective response, were downregulated in *Cort*^*−/−*^ cells in NX/NG, OGD-R or both (Fig. 7f)*.* On the contrary, upregulated genes in both physiological and injured *Cort*^*−/−*^ cells included *Ccl9*, *Il16*, *Lcp1*, *Cd93*, *Itga3* and *Itga4* integrins, and *Ulbp1*, all related to activation and chemoattraction of immune cells (Fig. 7f). However, genes required for the inflammatory-driven repair response, such as *Il33*, *Egr1*, *Gbp2,* *Gbp3* and *Il11,* were only upregulated in *Cort*^+*/*+^ cells after OGD-R conditions (Fig. 7f). None of these DEGs affecting immune pathways showed significant expression differences in *Cort*^*−/−*^ BECs during the progression from physiological to OGD-R states.

Notably, some hypoxia-dependent responsive genes with neuroprotective roles (such as *Egr1,* *Nr4a1,* *Fos,* *Gbp2,* *Mmp3,* *Spp1*, and *Nr1d1*) [34] were only upregulated in wild-type endothelium after ischemia and reperfusion (Fig. 7d, f, g). Instead, some of them (*Egr1,* *Nr4a1* and *Spp1*) were downregulated not only after OGD-R but also under NX/NG conditions in the absence of cortistatin (Fig. 7f, g). Similarly, genes whose expression regulates radical oxygen species formation were downregulated in *Cort*^−/−^ BECs even under NX/NG (Fig. 7g). Except for *Nos2* downregulation, none of these genes showed differential expression in *Cort*^*−/−*^ BECs dynamics from the healthy to the injured state. Intriguingly, we observed gene markers for epithelial cells (*Epcam*) and the keratinization process (Fig. 7h) among the top 25 upregulated DEGs in cortistatin-deficient brain endothelium incubated under OGD-R.

According to all these results, we observed that many components of crucial signalling networks involved in regulating endothelial cell biology were downregulated in the absence of cortistatin under physiological conditions (i.e*.,* genes involved in the regulation of Wnt, MAPK, TGF-β and Notch pathways) (Fig. 7i). In addition, various growth and transcription agents relevant for endothelium homeostasis were also downregulated in *Cort*^−/−^ BECs in NX/NG environment (Fig. 7h, i). Moreover, some kinases (*Mapk6,* *Ntrk3,* *Ddr2*) and phosphatases (*Ptprk*) were dysregulated in the absence of cortistatin. Conversely, some factors (*Igf1,* *Sox17,* *Pdgfb,* *Pdgfra*) were upregulated in physiological *Cort*^−/−^ cells, probably as a compensatory effect. Although some of the above-mentioned genes were also reduced in *Cort*^−/−^ BECs under OGD-R (Fig. 7i), none of them showed differences in the transition from a basal to an injured state neither in wild type nor in cortistatin-deficient cells.

Together, these results indicate that the lack of cortistatin induced downregulated gene networks not only in damaged but also in uninjured cells isolated from cortistatin-deficient mice. Besides, while *Cort*^+/+^ BECs appeared to achieve a canonical balanced response to damage upregulating genes linked to a further recovery response, these pathways were impaired in *Cort*^−/−^ BECs.

### Treatment with cortistatin protects brain endothelium integrity, regulates junction assembly and reverses immune activation

**Fig. 8:**
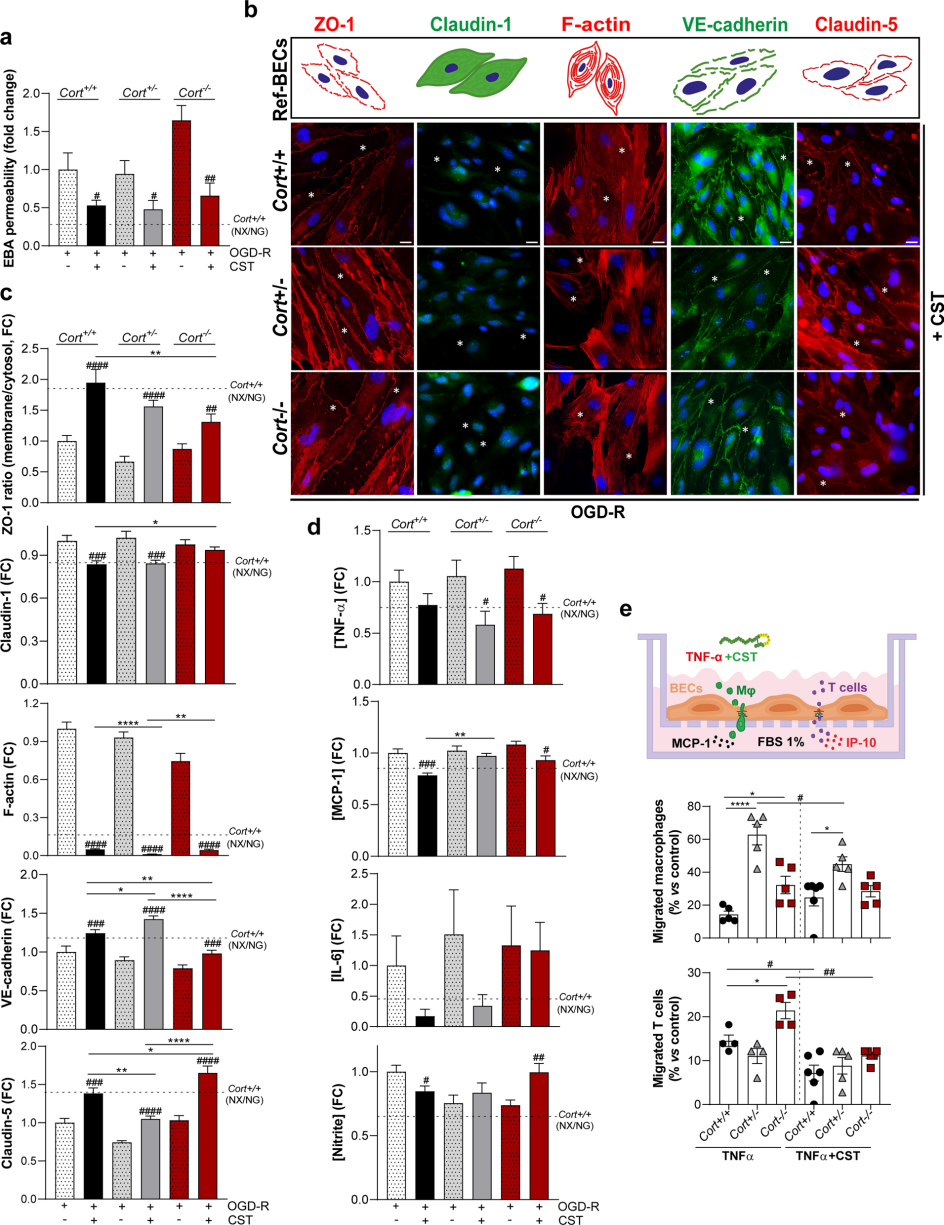
Cortistatin recovers the integrity and function of brain endothelial cells after ischemic-like conditions. **a** Endothelial permeability to EBA was represented as the tracer permeability fold change vs the permeability in OGD-R wild-type BECs (set at 1). *N* = 5–8 cultures/group. **b** Immunofluorescence analysis of cellular distribution for tight/adherens-junctions and stress fibers in wild-type and cortistatin-deficient BECs after OGD-R incubated with cortistatin. Asterisks indicate continuous membrane expression of ZO-1 (red), claudin-5 (red), and VE-cadherin (green), reduced intracellular claudin-1 (green), and random cytosolic F-actin organization (red), when compared to BECs exposed to OGD-R without cortistatin addition (as shown in Fig. 4b–d; schematic images with injury hallmarks represented in the top: Ref-BECs). Scale bar: 20 µm. **c** Quantification of location (ZO-1) and expression (claudin-1, claudin-5, VE-cadherin, F-actin) by fluorescence intensity analysed from 25–50 ROIs in 4 independent fields. *N* = 6 cultures/group. **d** Levels of inflammatory mediators determined in culture supernatants. *N* = 8 cultures/group. **e** *Top*, representation of transendothelial migration assay. Wild-type mice immune cells were incubated for 24 h at the top of *Cort*^+*/*+^, *Cort*^+/−^, and *Cort*^*−/−*^ BECs covered inserts, previously activated with TNF-α (10 ng/ml, 24 h). The migration assay was performed using MCP-1 (50 μg/ml) or IP-10 (50 μg/ml), as macrophages and T-cell chemoattractants, respectively. CST (100 nM) was applied when indicated. *Bottom*, data represented the percentage of migrated immune cells vs the control (empty-coated insert). *N* = 4–6 cultures/group. For data in c, d, FC represents fold change vs reference values based on OGD-R wild-type BECs quantification (set at 1). *Cort*^+*/*+^ BECs in NX/NG are also used as a basal condition reference (dashed line). Data are the mean ± SEM with dots representing individual values of independent cultures. Cells in each culture were derived from 4 pooled brains. *vs *Cort*^+*/*+^ BECs exposure to OGD-R + CST; ^#^vs BECs of corresponding genotype (*Cort*^+*/*+^, *Cort*^+/−^, *Cort*^*−/−*^) exposure to OGD-R.*^/#^*p* ≤ 0.05, **^/##^*p* ≤ 0.01, ***^/###^*p* ≤ 0.001, ****^/####^*p* ≤ 0.0001

To confirm the role of cortistatin as a controller of brain endothelium dynamics, we next investigated its effect in wild-type and cortistatin-deficient BECs. We showed that the addition of exogenous cortistatin after OGD and simultaneously to reoxygenation, in both control and cells with partial or complete absence of cortistatin, induced significant reduction in damage-derived endothelial permeability (Fig. 8a; Additional file 1: Fig. S10) that was coupled to the recovery of barrier permeability and of the functional intercellular architecture of the endothelium (Fig. 8b, c). This was demonstrated by the induction of the uniform and settled location of ZO-1 and claudin-5 in the peripheral membrane, the increased and solid expression of VE-cadherin, the decreased presence of cytosolic claudin-1, and the physiological reduced and randomised distribution of stress fibers (Fig. 8b, c). Moreover, the dysregulated immune response exerted by the injured endothelium was reversed to homeostatic state by cortistatin treatment (Fig. 8d). Specifically, TNF-α and MCP-1 levels were significantly reduced in *Cort*^+/+^*,* *Cort*^+/−^ and *Cort*^−/−^ BECs compared to the cytokine levels found in injured cells (Fig. 8d). Interestingly, exogenous cortistatin added to cortistatin-deficient endothelium significantly increased the reduced levels of nitrite after injury (Fig. 8d). Regarding the interaction between disrupted permeability, inflammatory endothelium, and cell migration, we next evaluated whether cortistatin could regulate immune trafficking throughout the wild-type and cortistatin-deficient brain endothelium (Fig. 8e). While transendothelial migration was enhanced in the absence of cortistatin, our results showed that incubation with cortistatin significantly modulated macrophages and T-cell displacement through activated brain endothelium (Fig. 8e).

**Fig. 9:**
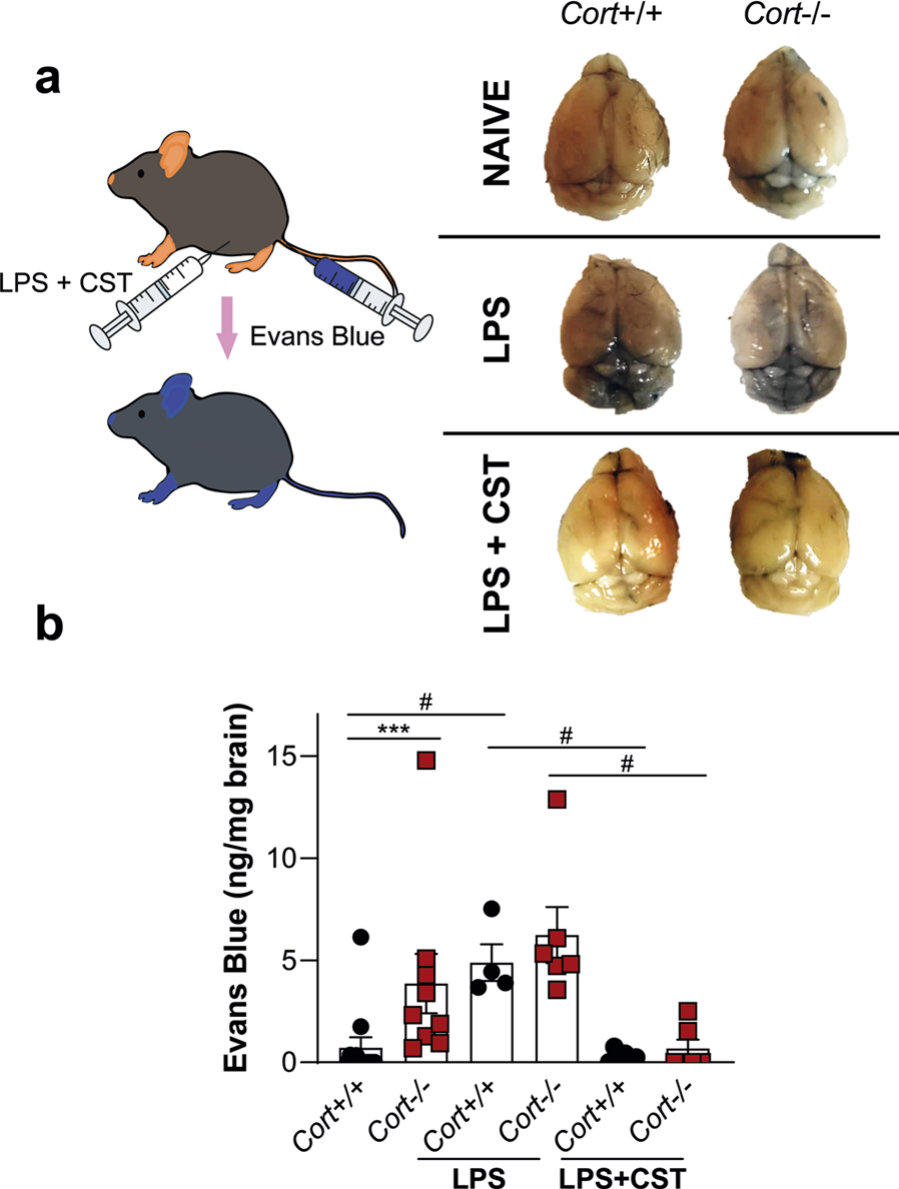
Administration of cortistatin reduces exacerbated murine brain endothelial barrier breakdown. **a** *Left*, wild-type and cortistatin-deficient mice (1 year) were injected intraperitoneally with LPS (6 mg/kg) for 6 h. Evans-Blue (2% in PBS) was injected through the tail vein 1 h before sacrifice. Cortistatin (1 nmol, in PBS) was intraperitoneally administered immediately after LPS. Control mice were injected with PBS as vehicle. *Right*, representative images show EB brain extravasation. **b** Brains were collected and minced in *N*,*N*-dimethylformamide at 55 ºC for 24 h. Supernatants were collected and EB content (ng/mg brain) was measured on a spectrophotometer at 620 nm. Data are the mean ± SEM with dots representing individual mice (*n* = 4–8/group). *p ≤ 0.05, **p ≤ 0.01

Despite endothelial cells being the main component of the neurovascular unit, astrocyte end-feet, microglia, pericytes, and other surrounding cells must also play important role in maintaining BBB homeostasis [1]. Following the data shown above, we analysed the potential role of cortistatin in the whole BBB structure. We found that systemic injection of cortistatin reversed the exacerbated leakage observed in wild-type and cortistatin-deficient mice subjected to mild neuroinflammation (Fig. 9). Notably, the lack of cortistatin resulted in enhanced permeability even in the absence of damage.

Altogether, these results suggest that endogenous cortistatin is critical for a healthy brain endothelial barrier and revealed the potential of this anti-inflammatory neuropeptide as a pleiotropic modulator of BBB dynamics.

## Discussion

**Fig. 10:**
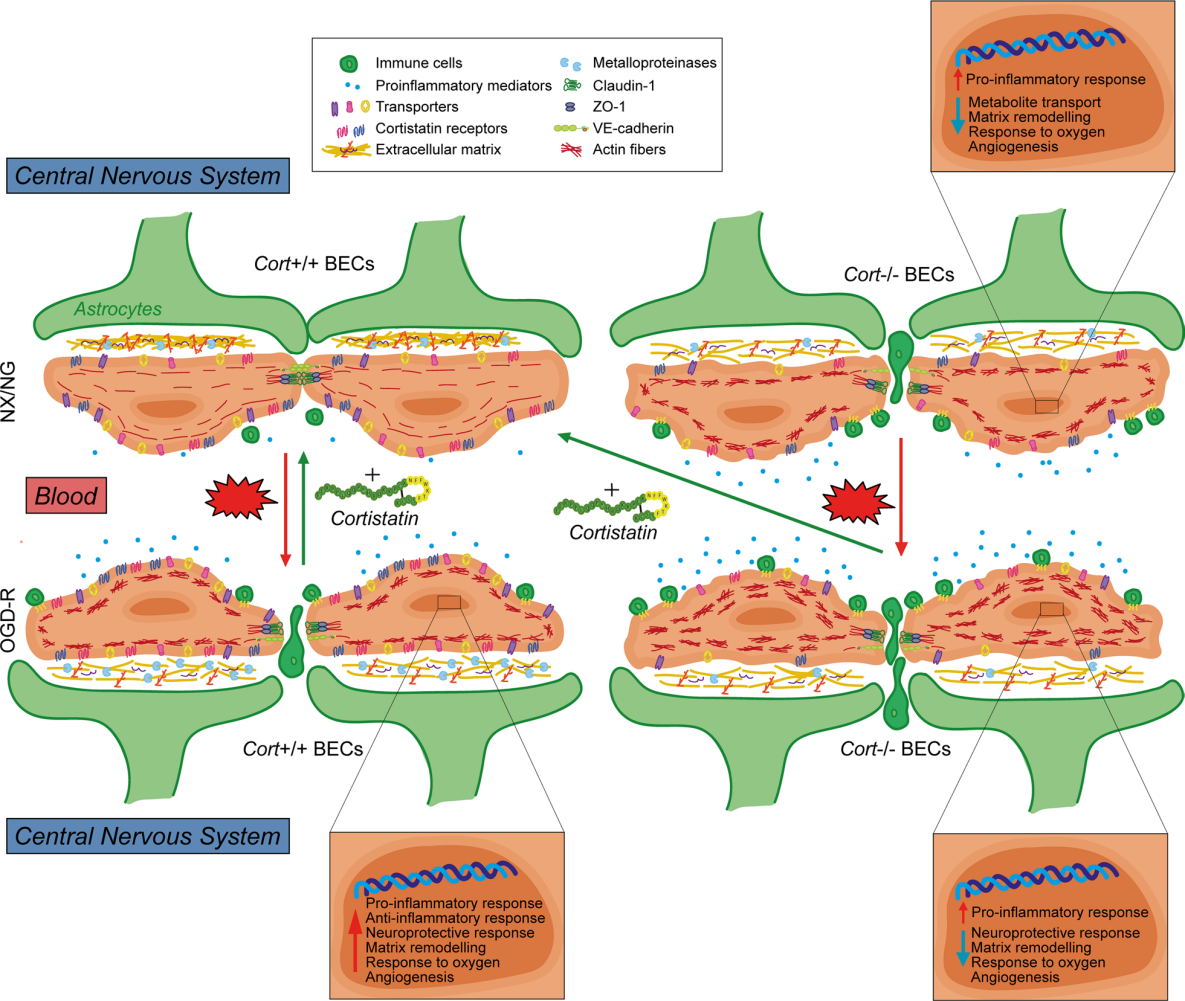
Schematic illustration of the proposed role of cortistatin in regulating physiology and pathology of brain endothelium. *Top* *left,* *Cort*^+*/*+^ BECs under normoxia/normoglycemia (NX/NG) conditions showing homeostatic endothelial barrier integrity. *Bottom* *left,* after oxygen–glucose deprivation and reoxygenation (OGD-R) to mimic ischemic damage (red arrow), *Cort*^+*/*+^ BECs suffered a canonical response (i.e., tight-junctions disruption, immune cell infiltration, proinflammatory mediators release, reorganization of actin in stress fibers, and ECM remodelling), combined with upregulation of protective and reparative transcriptional mechanisms (e.g., anti-inflammatory response or angiogenesis) (zoom for details). Remarkably, these changes were reversed with cortistatin treatment (green arrow). *Top* *right,* *Cort*^*−/−*^ BECs in NX/NG conditions presented similar phenotypic changes to *Cort*^+*/*+^ BECs in OGD-R (except for a decrease in the number of transporters and cortistatin receptors), but a different genetic programming (i.e., exacerbated immune response, deregulated ECM remodelling, and downregulation of angiogenesis and protective pathways related to the response to damage) (zoom for details). *Bottom* *right,* after exposing *Cort*^*−/−*^ BECs to OGD-R (red arrow), almost no significant transcriptional changes were observed when compared to *Cort*^*−/−*^ BECs in NX/NG. However, downregulation of metabolites transporters, exacerbated stress fiber formation, and increased immune activity was found. Cortistatin reverted these changes up to physiological conditions (*top* *left*, green arrow)

A tightly controlled BBB is essential for a fine-tuned microenvironment in the CNS, and its breakdown has been involved in several brain diseases. Therefore, research based on identifying BBB-related stabilizing factors focused on ECs, as the main BBB component, should be prioritized. To our knowledge, this is the first report to demonstrate the critical role of cortistatin as an endogenous factor in maintaining the integrity and functionality of the brain endothelium. We found that, even at low levels, cortistatin is produced by healthy murine and human brain endothelial cells, undergoing modulation after injury. Specifically, our findings demonstrated that the lack of cortistatin predisposes to endothelium disruption under basal conditions (Fig. 10). Notably, we observed that brain ECs isolated from heterozygous cortistatin mice, which secrete significant lower levels of cortistatin, exhibited phenotypic changes resembling those observed in wild-type endothelium after damage, where the levels of endogenous cortistatin are diminished. In addition, the breakdown of the brain endothelium from cortistatin-deficient mice was exacerbated after exposure to an ischemic-like context. Interestingly, the exogenous addition of cortistatin reversed hallmark features of the disturbed endothelium, such as intercellular junction instability, increased paracellular and transendothelial permeability, and dysregulated endothelial immune activity (Fig. 10). Based on these findings, we propose the involvement of various non-excluding and complementary mechanisms that could explain the role of cortistatin in BBB stability.

Regarding brain barrier integrity, numerous studies have demonstrated that disturbances in the content, cellular distribution, and/or post-translational modifications of TJs and AJs in ECs, increase BBB abnormalities and initiate neurodegenerative diseases [1, 37]. Recently, in addition to the well-known roles of TJs and AJs in organizing cell architecture, polarity, and cell–cell contacts, other non-canonical functions, including cell proliferation, differentiation, participation in angiogenesis and inflammatory processes, and regulation of gene expression, have also been demonstrated [4, 38]. Accordingly, we found an induction of hyperpermeability in the endothelium lacking cortistatin, which was probably accompanied by the alteration of both barrier and non-barrier roles of the TJ/AJs. In particular, the expression of some TJ/AJ genes (*Jam2,* *Gja1,* *Pcdh18,* *Pcdh19,* *Cdh11*) crucial for connecting adjacent ECs [3, 39], was downregulated. On the contrary, levels of *Cldn1,* *Cldn4,* *Cldn6,* *Cldn9,* *Cgn,* *F11r,* and *Cdh1*, associated with exacerbated inflammation, perturbation of the TJ assembly [27, 40], and even with mesenchymal transformation or tumour progression [41, 42], were overexpressed. Importantly, cortistatin deficiency altered protein levels and cell distribution of ZO-1, claudin-1, claudin-5, and VE-cadherin, without significantly affecting their transcript levels. In fact, different studies have shown that while different insults may not impact TJ expression, they can affect their cellular redistribution and functional organization. For instance, ZO-1 translocation from the membrane out to the cytosol, without alterations in its expression, affected barrier integrity and resulted in TJ disorganization [43].

On the other hand, although controversial findings about the role of claudin-1 have been reported [40, 44, 45], several studies indicate that claudin-1 increases in the disrupted endothelium, affects claudin-5 assembly, and its cytosolic accumulation has been correlated with TJ arrangement deficits and limited full recovery of the barrier [27, 46]. In addition, emerging evidence suggests that claudin proteins can localize to sites outside of the tight-junction complex. Indeed, claudin-1 seems to be representative of claudins that shuttle between the cytoplasm and nucleus, acquiring non-canonical functions, including cell proliferation, differentiation, participation in angiogenesis and inflammatory processes, and regulation of gene expression [38, 47]. In fact, reducing claudin-1 was beneficial for modulating BBB permeability and endothelial inflammatory phenotype [27]. Therefore, the disruptive expression of ZO-1, VE-cadherin, and claudin-5, along with the cytosolic overexpression of ZO-1 and claudin-1 in cortistatin-deficient ECs (Fig. 10), in both injury and physiological conditions, emphasizes the relevance of this neuropeptide in maintaining a properly sealed and healthy barrier. In addition, TJs also regulate the distribution of enzymes and receptors in the luminal and abluminal membrane domains [39, 48]. Indeed, the disruption of junctional proteins in the absence of cortistatin was accompanied by the loss of cell polarity and downregulation of several transporters involved in endothelial metabolic support (Fig. 10), which can also have a negative impact on BBB permeability [1].

Furthermore, TJs are connected to actin cytoskeleton through intracellular proteins, providing a dynamic organization of actin filaments, crucial for BBB integrity. In this sense, the reorganization of actin filaments into stress fibers is often associated with TJ instability, endothelial barrier dysfunction, and hyperpermeability [49]. In addition to TJ disruption, endothelium from cortistatin-deficient mice also exhibited an increase in F-actin stress fiber formation, which was correlated with a marked cytoskeletal disassembly in both uninjured and injured conditions (Fig. 10). In this context, both hyperpermeability and cytoskeleton disorganization in the absence of cortistatin may be linked to some dysregulated pathways involving kinases/phosphatases (such as *Mapk6,* *Dmpk,* *Ntrk3* and *Ptprk*), which have been described as crucial regulators of TJ/AJs organization/dissociation [50].

Moreover, ECM plays a key role in maintaining BBB dynamics. Alterations in ECM can result in BBB leakage, as evidenced by the decreased expression of certain ECM components, such as collagens and laminins, in aged mice [2]. Besides, mutations in genes related to collagens have been associated with leaky vessels that predispose to haemorrhage in humans [51]. Notably, we observed that the majority of genes involved in ECM composition were downregulated in cortistatin-deficient endothelium. Besides its physical and mechanical properties, ECM is crucial for matrix-cell and cell–cell signalling, with integrins complexes acting as the main receptors. An unbalanced ratio of ECM components and/or dysfunction of integrins can lead to BBB abnormalities as multiple endothelial functions are affected (survival, migration, differentiation, and cell adhesion) [52]. Alternatively, ECM components undergo alterations and modifications driven by matrix metalloproteinases (MMPs) and metallopeptidases, which are necessary to induce angiogenesis and vascular remodelling after injury [53, 54]. In this regard, while wild-type endothelial cells exhibited a canonical balanced response to damage by upregulating the pathways linked to recovery (including ECM reorganization, angiogenesis, cell fate determination, and response to hypoxia), these responses were impaired in *Cort*^−/−^ BECs (Fig. 10).

Unexpectedly, we found high expression of the epithelial markers *Krt7,* *Krt8*, *Krt18*, *Cdh1,* and *Epcam* in cortistatin-deficient BECs. These are signature genes for choroid plexus epithelial cells [55], which can contaminate primary isolates of brain microvascular ECs. However, differentially increased expression of these factors was only identified in the cortistatin-deficient injured endothelium. This finding suggests that endogenous cortistatin may play a role in promoting endothelial cell determination over an epithelial-like fate. Indeed, regulatory factors of endothelial cell fate specification were downregulated in the endothelium lacking cortistatin. Confirming this, recent data also demonstrated an endogenous role for cortistatin as a controller of tissue-specific cell commitment in fibrogenic responses [56].

On the other side, it has been described the participation of IL-6, TNF-α, and MCP-1 in the loss of BBB integrity, stress fiber formation, TJ remodelling, and immune cell infiltration (reviewed in [57, 58]). Our data revealed a dysregulated immune response in the brain endothelium lacking cortistatin, characterized by the production of inflammatory factors, enhanced immune cell migration, inhibited immunoregulatory and restorative pathways, and upregulated integrin signalling. Despite other studies found high levels of peripheral glucocorticoids in cortistatin-deficient mice [7, 11], suggesting an immune suppressive phenotype in these animals, in this study the inflammatory phenotype of CST-deficient cells suggests that none of the protective effects associated with glucocorticoids are observed in this context. In fact, *Anxa1*, a glucocorticoid-induced endothelial factor correlated with BBB disruption [59], was downregulated in the absence of cortistatin.

Surprisingly, we observed reduced nitrite levels in *Cort*^*−/−*^ BECs. Nitric oxide has been associated with both protective and deleterious effects, but recent reports have confirmed that insufficient availability of endothelial nitric oxide leads to endothelial dysfunction [60]. Importantly, the balance between the activities Nos2 and Nos3, along with the scavenging function of the superoxide anion, can regulate nitric oxide levels [60]. Future studies will address whether these pathways can be cooperating to reduce nitric oxide when cortistatin is absent.

Besides this, from a molecular perspective, our study revealed the downregulation of several gene networks involved in matrix remodelling, cytoskeleton architecture, junctions assembly, endothelial commitment, and immune activity (reviewed in [4]) in cortistatin-deficient mice (Fig. 10). Among the affected pathways, we identified VEGF-VEGFR2-Nrp1, TGF-β-Nrp1 and Notch signalling (all essential for angiogenesis), Wnt pathway (critical for BBB maturation and barriergenesis), Ang1/Tie2 interaction (essential for stabilizing TJs), and semaphorins signalling (key in cerebral vasculature growth and BBB functions). Although further research is needed to investigate the specific contribution of cortistatin in each network, our results provide support for the key role of endogenous cortistatin in the fine regulation of these interconnected pathways. Moreover, we found that several markers of neurodegeneration and aging (*Capn11,* *Eno1b,* *Cmpk2,* *Galnt3,* and *Ubiad1*, among others) were upregulated in cortistatin-deficient endothelium. Recently, the impact of normal ageing on BBB integrity and plasticity has been demonstrated [61]. Beyond ageing, other biological processes, such as sleep behaviour, can influence BBB functions [62, 63]. In this sense, cortistatin has been identified as a crucial regulator of sleep homeostasis through activity-dependent BDNF [64, 65]. Interestingly, we found a decreased expression of *Bdnf* in cortistatin-deficient brain endothelium, supporting a link between BDNF, cortistatin and sleep physiology.

Finally, from a therapeutic point of view, our results suggest that the beneficial role of cortistatin could be exerted on brain endothelial cells in an autocrine/paracrine manner. While protective effects of somatostatin, ghrelin, and receptors-agonists in the endothelium have been reported [24, 26, 66], controversial findings regarding the pro- and anti-angiogenic properties of ghrelin [24, 67] and somatostatin-induced hyperpermeability have been observed [68]. However, similar studies have never been conducted with cortistatin. As previously described, cortistatin can exert many different physiological roles compared to somatostatin and ghrelin [7, 8, 69]. The activation of somatostatin/ghrelin receptors can trigger diverse signalling pathways [70–72]. Probably, the potential capability of cortistatin to synergistically signal through somatostatin/ghrelin receptors (in addition to a yet unknown-specific receptor) could imply an advantage vs the individual regulation of the BBB by somatostatin/ghrelin. Specifically, while somatostatin inhibits cAMP accumulation, cortistatin has been reported to stimulate it [73]. Interestingly, increased levels of cAMP are associated with greater endothelial TJ integrity [74], cytoskeleton and actin filaments stabilization, reinforcement of cell–matrix interactions [75], and anti-inflammatory actions [8, 76]. Besides, evidence supports that cortistatin promotes peripheral protective vascular responses after injury via somatostatin/ghrelin receptors, by inducing cAMP and inhibiting calcium rise [15]. In addition, it was described that the combined activation of SSTR5 and ghrelin receptor greatly increased cAMP accumulation, suggesting intracellular signal convergence and/or receptors interaction [77]. In our study, we found that *Sstr4* and *Sstr2* were differentially regulated comparing wild-type and cortistatin-deficient cells in physiological and pathological conditions. While future experiments would be necessary to characterize cortistatin-induced intracellular pathways, we advocate that the binding of cortistatin to these receptors might be regulating brain endothelium integrity. Alternatively, since somatostatin and ghrelin receptors are involved in the immunoregulatory activity of cortistatin [5], we cannot discard that the anti-inflammatory properties of cortistatin can be contributing to the protection of barrier integrity. Altogether, our results showing the efficient recovery of endothelium integrity after cortistatin treatment, support the capacity of this neuropeptide to directly limit injured ischemic-like responses. However, considering the in vivo protective effects of cortistatin on BBB leakage, and the expression of cortistatin and its receptors [11] in other BBB components (i.e., astrocytes, pericytes, and microglia), we can hypothesize that cortistatin might also influence brain endothelium dynamics through other glial cells. Moreover, we cannot discard that BBB-penetrating peripheral factors from cortistatin-deficient mice and/or surrounding cells lacking cortistatin could have influenced and determined endothelial cell phenotype.

In conclusion, our study contributes to the evolving understanding of the BBB as a highly dynamic and plastic structure that allows communication between the blood and the CNS, rather than being the traditionally considered impermeable wall [78]. We recognize that neurodegenerative/neuroinflammatory disorders, such as stroke, multiple sclerosis, or Alzheimer´s disease, often present acute, subacute, and chronic phases with different grades of BBB permeability. Therefore, it is crucial to consider approaches that finely modulate permeability alterations beyond simplistic categorizations of BBB opening or closing as solely "good" or "bad”. In this sense, our models not only focus on the pathological and acute event of ischemia but also considered subsequent responses leading to repair and recovery during reoxygenation. In this regard, our study has several implications. From a physiological point of view, we uncovered a new role for endogenous cortistatin in maintaining BBB physiological functions and in recovering integrity after injury. Moreover, our findings emphasize the essential interplay between signals from the CNS and the periphery, exemplified by endogenous modulation of cortistatin levels, for maintaining a healthy BBB. We have shown for the first time that BBB is markedly impaired in the brains of cortistatin-deficient mice, which makes them susceptible to further disruptive changes. Indeed, lack of cortistatin confers the endothelium a pre-existing dysregulated phenotype, which could lead to a deactivated and/or quiescent non-responding behaviour upon further damage. Alternatively, from a treatment perspective, our findings support the beneficial effect of exogenous cortistatin by rescuing defects in BBB function. Given the scarce number of therapeutic agents available to restore BBB function in neurodegeneration [79, 80], the use of multifactorial agents that target at the same time components of the TJ complex, modulate endothelial permeability at different grades, and control immune dysregulation, looks promising for the treatment and prevention of neuroinflammatory/neurodegenerative disorders. Furthermore, restoring BBB disruption would also address the everlasting challenge of drug delivery to the injured brain, since vascular changes and accumulation of blood-derived toxics often restrict the transport of drugs to the brain [4].

In summary, the data presented in this study underscore the key role of cortistatin as a pleiotropic agent that mediates anti-inflammatory endothelial functions and exerts BBB reparative properties, through organizing endothelial junction proteins and reducing immune exacerbated responses (Fig. 10). Our findings also highlight the brain endothelium as an important but neglected source of molecular targets that should be particularly investigated. Overall, understanding the role of cortistatin in the physiology of the cerebral microvasculature has broader implications for gaining insights into the involvement of BBB disruption in various CNS disorders. The knowledge obtained from this study may contribute to the development of novel therapeutic strategies aimed at preserving BBB integrity and ameliorating CNS pathologies associated with BBB dysfunction.

### Supplementary Information

**Additional file 1**: Extended methods for animals, b.End5 and BECs cells, human BBB model, endothelial permeability assay, immunocytochemistry, determination of inflammatory factors, transendothelial migration assay, in vivo BBB permeability assay, RNA extraction and determination of gene expression, next-generation transcriptome sequencing. **Table S1**. Sequence of primers used for qPCR dynamic array. **Table S2**. Sequence of primers used for real-time PCR quantifications. **Figure S1**. Principal coordinates analysis (PCoA) and unsupervised hierarchical clustering of normalized RNA-Seq data. **Figure S2**. Expression profile of the components of the cortistatin pathway in b.End5 cells. **Figure S3**. Cortistatin modulates the immune function of activated b.End5 cells. **Figure S4**. Expression profile of the components of the cortistatin pathway in murine brain endothelial cells. **Figure S5**. Characterization of cortistatin in human brain endothelium **Figure S6**. Lack of barrier integrity and altered expression profile in cortistatin-deficient cells. **Figure S7**. Analysis of specific markers in BECs and validation of gene expression. **Figure S8**. Characterization of gene pathways in wild type vs cortistatin-deficient brain endothelium. **Figure S9**. Characterization of gene pathways in the dynamics of brain endothelium. **Figure S10**. Cortistatin restores the integrity of injured brain endothelial cells.

**Additional file 2**: **Table S3**. Differentially expressed genes (DEGs) in *Cort*^−/−^  BECs in NX/NG compared to *Cort*^+/+^ BECs. **Table S4**. Differentially expressed genes (DEGs) in *Cort*^−/−^ BECs in OGD-R compared to *Cort*^+/+^ BECs. **Table S5**. DEGs for NX/NG-incubated *Cort*^−/−^ vs *Cort*^+/+^ BECs included in Gene Ontology (GO) terms. **Table S6**. DEGs for OGD-R-incubated *Cort*^−/−^ vs *Cort*^+/+^ BECs included in Gene Ontology (GO) terms. **Table S7**. Differentially expressed genes (DEGs) in the dynamics of *Cort*^+/+^ BECs from NX/NG to OGD-R. **Table S8**. Differentially expressed genes (DEGs) in the dynamics of *Cort*^−/−^ BECs from NX/NG to OGD-R. **Table S9**. DEGs for *Cort*^+/+^ BECs from NX/NG to OGD-R included in Gene Ontology (GO) terms. **Table S10**. DEGs for *Cort*^−/−^ BECs from NX/NG to OGD-R included in Gene Ontology (GO) terms. **Table S11**. Comparative values between log2FC and RPKM for differentially expressed genes (DEGs) depicted in Fig. 7.

## Data Availability

RNA-seq data sets generated during the current study are available in the GEO repository under accession number GSE207405, https://www.ncbi.nlm.nih.gov/geo/query/acc.cgi?acc=GSE207405.

## Abbreviations

AJ: Adherens-junctions
BBB: Blood–brain barrier
BECs: Brain endothelial cells
CNS: Central nervous system
CST: Cortistatin
DEGs: Differential-expressed genes
EBA: Evans blue-albumin
ECs: Endothelial cells
GD: Glucose deprivation
hBLECs: Human brain-like endothelial cells
HPX: Hypoxia
HPX-R: Hypoxia-reoxygenation
IL-6: Interleukin-6
LPS: Lipopolysaccharide inflammation
MCP-1: Monocyte chemoattractant protein-1
NaF: Sodium fluorescein
NX/NG: Normoxia–normoglycemia
OGD-R: Oxygen–glucose deprivation and reoxygenation
SST: Somatostatin
TJ: Tight-junctions
TNF-α: Tumor necrosis factor-alpha
ZO-1: Zonula occludens-1

## References

[1] Keaney J, Campbell M (2015). The dynamic blood–brain barrier. FEBS J.

[2] Reed MJ, Damodarasamy M, Banks WA (2019). The extracellular matrix of the blood–brain barrier: structural and functional roles in health, aging, and Alzheimer’s disease. Tissue Barriers.

[3] Cong X, Kong W (2020). Endothelial tight junctions and their regulatory signaling pathways in vascular homeostasis and disease. Cell Signal.

[4] Sweeney MD, Zhao Z, Montagne A, Nelson AR, Zlokovic BV (2019). Blood–brain barrier: from physiology to disease and back. Physiol Rev.

[5] Delgado M, Gonzalez-Rey E. Role of cortistatin in the stressed immune system. In: Savino W, Guaraldi F, editors. Frontiers of hormone research. 2017; p. 110–20. Available from: https://www.karger.com/Article/FullText/452910. 10.1159/000452910 28245456

[6] de Lecea L, Criado JR, Prospero-Garcia O, Gautvik KM, Schweitzer P, Danielson PE (1996). A cortical neuropeptide with neuronal depressant and sleep-modulating properties. Nature.

[7] Córdoba-Chacón J, Gahete MD, Pozo-Salas AI, Martínez-Fuentes AJ, de Lecea L, Gracia-Navarro F (2011). Cortistatin is not a somatostatin analogue but stimulates prolactin release and inhibits GH and ACTH in a gender-dependent fashion: potential role of ghrelin. Endocrinology.

[8] Deghenghi R, Papotti M, Ghigo E, Muccioli G (2001). Cortistatin, but not somatostatin, binds to growth hormone secretagogue (GHS) receptors of human pituitary gland. J Endocrinol Invest.

[9] Braun H, Schulz S, Becker A, Schröder H, Höllt V (1998). Protective effects of cortistatin (CST-14) against kainate-induced neurotoxicity in rat brain. Brain Res.

[10] Chiu C-T, Wen L-L, Pao H-P, Wang J-Y (2011). Cortistatin is induced in brain tissue and exerts neuroprotection in a rat model of bacterial meningoencephalitis. J Infect Dis.

[11] Souza-Moreira L, Morell M, Delgado-Maroto V, Pedreño M, Martinez-Escudero L, Caro M (2013). Paradoxical effect of cortistatin treatment and its deficiency on experimental autoimmune encephalomyelitis. J Immunol.

[12] Falo CP, Benitez R, Caro M, Morell M, Forte-Lago I, Hernandez-Cortes P (2021). The neuropeptide cortistatin alleviates neuropathic pain in experimental models of peripheral nerve injury. Pharmaceutics.

[13] Delgado-Maroto V, Benitez R, Forte-Lago I, Morell M, Maganto-Garcia E, Souza-Moreira L (2017). Cortistatin reduces atherosclerosis in hyperlipidemic ApoE-deficient mice and the formation of foam cells. Sci Rep.

[14] Delgado-Maroto V, Falo CP, Forte-Lago I, Adan N, Morell M, Maganto-Garcia E (2017). The neuropeptide cortistatin attenuates experimental autoimmune myocarditis via inhibition of cardiomyogenic T cell-driven inflammatory responses. Br J Pharmacol.

[15] Duran-Prado M, Morell M, Delgado-Maroto V, Castaño JP, Aneiros-Fernandez J, de Lecea L (2013). Cortistatin inhibits migration and proliferation of human vascular smooth muscle cells and decreases neointimal formation on carotid artery ligation. Circ Res.

[16] Yang T, Roder KE, Abbruscato TJ (2007). Evaluation of bEnd5 cell line as an in vitro model for the blood–brain barrier under normal and hypoxic/aglycemic conditions. J Pharm Sci.

[17] Welser-Alves JV, Boroujerdi A, Milner R, Milner R (2014). Isolation and culture of primary mouse brain endothelial cells. Cerebral angiogenesis: methods and protocols.

[18] Cecchelli R, Aday S, Sevin E, Almeida C, Culot M, Dehouck L (2014). A stable and reproducible human blood–brain barrier model derived from hematopoietic stem cells. PLoS ONE.

[19] Takata F, Dohgu S, Yamauchi A, Matsumoto J, Machida T, Fujishita K (2013). In vitro blood–brain barrier models using brain capillary endothelial cells isolated from neonatal and adult rats retain age-related barrier properties. PLoS ONE.

[20] Jangula A, Murphy EJ (2013). Lipopolysaccharide-induced blood brain barrier permeability is enhanced by alpha-synuclein expression. Neurosci Lett.

[21] Fuentes-Fayos AC, Vázquez-Borrego MC, Jiménez-Vacas JM, Bejarano L, Pedraza-Arévalo S, López LF (2020). Splicing machinery dysregulation drives glioblastoma development/aggressiveness: oncogenic role of SRSF3. Brain.

[22] Ruiz JL, Terrón-Camero LC, Castillo-González J, Fernández-Rengel I, Delgado M, Gonzalez-Rey E (2023). reanalyzerGSE: tackling the everlasting lack of reproducibility and reanalyses in transcriptomics. bioRxiv.

[23] Andrés-León E, Rojas AM (2019). miARma-Seq, a comprehensive pipeline for the simultaneous study and integration of miRNA and mRNA expression data. Methods.

[24] Li R, Yao G, Zhou L, Zhang M, Yan J (2022). The ghrelin-GHSR-1a pathway inhibits high glucose-induced retinal angiogenesis in vitro by alleviating endoplasmic reticulum stress. Eye Vis.

[25] Yan S, Li M, Chai H, Yang H, Lin PH, Yao Q (2005). TNF-α decreases expression of somatostatin, somatostatin receptors, and cortistatin in human coronary endothelial cells. J Surg Res.

[26] Basivireddy J, Somvanshi RK, Romero IA, Weksler BB, Couraud P-O, Oger J (2013). Somatostatin preserved blood brain barrier against cytokine induced alterations: possible role in multiple sclerosis. Biochem Pharmacol.

[27] Sladojevic N, Stamatovic SM, Johnson AM, Choi J, Hu A, Dithmer S (2019). Claudin-1-dependent destabilization of the blood–brain barrier in chronic stroke. J Neurosci.

[28] Coisne C, Dehouck L, Faveeuw C, Delplace Y, Miller F, Landry C (2005). Mouse syngenic in vitro blood–brain barrier model: a new tool to examine inflammatory events in cerebral endothelium. Lab Invest.

[29] Kuntz M, Mysiorek C, Pétrault O, Boucau M-C, Aijjou R, Uzbekov R (2014). Transient oxygen–glucose deprivation sensitizes brain capillary endothelial cells to rtPA at 4h of reoxygenation. Microvasc Res.

[30] Gerhartl A, Pracser N, Vladetic A, Hendrikx S, Friedl H-P, Neuhaus W (2020). The pivotal role of micro-environmental cells in a human blood–brain barrier in vitro model of cerebral ischemia: functional and transcriptomic analysis. Fluids Barriers CNS.

[31] Gray KM, Jung JW, Inglut CT, Huang H-C, Stroka KM (2020). Quantitatively relating brain endothelial cell–cell junction phenotype to global and local barrier properties under varied culture conditions via the Junction Analyzer Program. Fluids Barriers CNS.

[32] Babinska A, Kedees MH, Athar H, Ahmed T, Batuman O, Ehrlich YH (2002). F11-receptor (F11R/JAM) mediates platelet adhesion to endothelial cells: role in inflammatory thrombosis. Thromb Haemost.

[33] Beard RS, Haines RJ, Wu KY, Reynolds JJ, Davis SM, Elliott JE (2014). Non-muscle Mlck is required for β-catenin- and FoxO1-dependent downregulation of Cldn5 in IL-1β-mediated barrier dysfunction in brain endothelial cells. J Cell Sci.

[34] Sperandio S, Fortin J, Sasik R, Robitaille L, Corbeil J, de Belle I (2009). The transcription factor Egr1 regulates the HIF-1α gene during hypoxia. Mol Carcinog.

[35] Kerr DJ, Marsillo A, Guariglia SR, Budylin T, Sadek R, Menkes S (2016). Aberrant hippocampal Atp8a1 levels are associated with altered synaptic strength, electrical activity, and autistic-like behavior. Biochim Biophys Acta BBA Mol Basis Dis..

[36] Zhao Z, Nelson AR, Betsholtz C, Zlokovic BV (2015). Establishment and dysfunction of the blood–brain barrier. Cell.

[37] Zlokovic BV (2008). The blood–brain barrier in health and chronic neurodegenerative disorders. Neuron.

[38] Hagen SJ (2017). Non-canonical functions of claudin proteins: beyond the regulation of cell–cell adhesions. Tissue Barriers.

[39] Tietz S, Engelhardt B (2015). Brain barriers: crosstalk between complex tight junctions and adherens junctions. J Cell Biol.

[40] Schlingmann B, Overgaard CE, Molina SA, Lynn KS, Mitchell LA, Dorsainvil White S (2016). Regulation of claudin/zonula occludens-1 complexes by hetero-claudin interactions. Nat Commun.

[41] Yan T, Tan Y, Deng G, Sun Z, Liu B, Wang Y (2022). TGF-β induces GBM mesenchymal transition through upregulation of CLDN4 and nuclear translocation to activate TNF-α/NF-κB signal pathway. Cell Death Dis.

[42] Zavala-Zendejas VE, Torres-Martinez AC, Salas-Morales B, Fortoul TI, Montaño LF, Rendon-Huerta EP (2011). Claudin-6, 7, or 9 overexpression in the human gastric adenocarcinoma cell line AGS increases its invasiveness, migration, and proliferation rate. Cancer Invest.

[43] Sajja RK, Prasad S, Cucullo L (2014). Impact of altered glycaemia on blood–brain barrier endothelium: an in vitro study using the hCMEC/D3 cell line. Fluids Barriers CNS.

[44] Liebner S, Fischmann A, Rascher G, Duffner F, Grote E-H, Kalbacher H (2000). Claudin-1 and claudin-5 expression and tight junction morphology are altered in blood vessels of human glioblastoma multiforme. Acta Neuropathol.

[45] Pfeiffer F, Schäfer J, Lyck R, Makrides V, Brunner S, Schaeren-Wiemers N (2011). Claudin-1 induced sealing of blood–brain barrier tight junctions ameliorates chronic experimental autoimmune encephalomyelitis. Acta Neuropathol.

[46] Gonçalves A, Ambrósio AF, Fernandes R (2013). Regulation of claudins in blood–tissue barriers under physiological and pathological states. Tissue Barriers.

[47] Alshbool Fatima Z, Mohan S (2014). Emerging multifunctional roles of claudin tight junction proteins in bone. Endocrinology.

[48] Haseloff RF, Dithmer S, Winkler L, Wolburg H, Blasig IE (2015). Transmembrane proteins of the tight junctions at the blood–brain barrier: structural and functional aspects. Semin Cell Dev Biol.

[49] Lai C-H, Kuo K-H, Leo JM (2005). Critical role of actin in modulating BBB permeability. Brain Res Rev.

[50] Rao RK, Basuroy S, Rao VU, Karnaky KJ, Gupta A (2002). Tyrosine phosphorylation and dissociation of occludin–ZO-1 and E-cadherin–β-catenin complexes from the cytoskeleton by oxidative stress. Biochem J.

[51] Gould DB, Phalan FC, van Mil SE, Sundberg JP, Vahedi K, Massin P (2006). Role of COL4A1 in small-vessel disease and hemorrhagic stroke. N Engl J Med.

[52] Baeten KM, Akassoglou K (2011). Extracellular matrix and matrix receptors in blood–brain barrier formation and stroke. Dev Neurobiol.

[53] Bauer AT, Bürgers HF, Rabie T, Marti HH (2010). Matrix metalloproteinase-9 mediates hypoxia-induced vascular leakage in the brain via tight junction rearrangement. J Cereb Blood Flow Metab.

[54] Ma Z, Mao C, Jia Y, Fu Y, Kong W (2020). Extracellular matrix dynamics in vascular remodeling. Am J Physiol Cell Physiol.

[55] Ghersi-Egea J-F, Strazielle N, Catala M, Silva-Vargas V, Doetsch F, Engelhardt B (2018). Molecular anatomy and functions of the choroidal blood–cerebrospinal fluid barrier in health and disease. Acta Neuropathol.

[56] Benitez R, Caro M, Andres-Leon E, O’Valle F, Delgado M (2022). Cortistatin regulates fibrosis and myofibroblast activation in experimental hepatotoxic- and cholestatic-induced liver injury. Br J Pharmacol.

[57] Sonar SA, Lal G (2018). Blood–brain barrier and its function during inflammation and autoimmunity. J Leukoc Biol.

[58] de Vries HE, Blom-Roosemalen MCM, van Oosten M, de Boer AG, van Berkel TJC, Breimer DD (1996). The influence of cytokines on the integrity of the blood-brain barrier in vitro. J Neuroimmunol.

[59] Cristante E, McArthur S, Mauro C, Maggioli E, Romero IA, Wylezinska-Arridge M (2013). Identification of an essential endogenous regulator of blood–brain barrier integrity, and its pathological and therapeutic implications. Proc Natl Acad Sci USA.

[60] Pacher P, Beckman JS, Liaudet L (2007). Nitric oxide and peroxynitrite in health and disease. Physiol Rev.

[61] Farrall AJ, Wardlaw JM (2009). Blood–brain barrier: ageing and microvascular disease—systematic review and meta-analysis. Neurobiol Aging.

[62] He J, Hsuchou H, He Y, Kastin AJ, Wang Y, Pan W (2014). Sleep restriction impairs blood–brain barrier function. J Neurosci.

[63] Sun J, Wu J, Hua F, Chen Y, Zhan F, Xu G (2020). Sleep Deprivation Induces Cognitive Impairment by Increasing Blood-Brain Barrier Permeability via CD44. Front Neurol.

[64] Bourgin P, Fabre V, Huitrón-Reséndiz S, Henriksen SJ, Prospero-Garcia O, Criado JR (2007). Cortistatin promotes and negatively correlates with slow-wave sleep. Eur J Neurosci.

[65] Faraguna U, Vyazovskiy VV, Nelson AB, Tononi G, Cirelli C (2008). A causal role for brain-derived neurotrophic factor in the homeostatic regulation of sleep. J Neurosci.

[66] Monte MD, Cammalleri M, Martini D, Casini G, Bagnoli P (2007). Antiangiogenic role of somatostatin receptor 2 in a model of hypoxia-induced neovascularization in the retina: results from transgenic mice. Invest Ophthalmol Vis Sci.

[67] Zaniolo K, Sapieha P, Shao Z, Stahl A, Zhu T, Tremblay S (2011). Ghrelin modulates physiologic and pathologic retinal angiogenesis through GHSR-1a. Invest Ophthalmol Vis Sci.

[68] Aslam M, Idrees H, Ferdinandy P, Helyes Z, Hamm C, Schulz R (2022). Somatostatin primes endothelial cells for agonist-induced hyperpermeability and angiogenesis in vitro. Int J Mol Sci.

[69] Spier AD, de Lecea L (2000). Cortistatin: a member of the somatostatin neuropeptide family with distinct physiological functions. Brain Res Rev.

[70] Córdoba-Chacón J, Gahete MD, Duran-Prado M, Pozo-Salas AI, Malagón MM, Gracia-Navarro F (2010). Identification and characterization of new functional truncated variants of somatostatin receptor subtype 5 in rodents. Cell Mol Life Sci.

[71] Durán-Prado M, Malagón MM, Gracia-Navarro F, Castaño JP (2008). Dimerization of G protein-coupled receptors: new avenues for somatostatin receptor signalling, control and functioning. Mol Cell Endocrinol.

[72] Jiang H, Smith R. Modification of ghrelin and somatostatin signaling by formation of GHSR-1a/SSTR5 heterodimers. The Endocrine Society’s 89th Annual Meeting. 2007;P9: 299.

[73] Sánchez-Alavez M, Gómez-Chavarin M, Navarro L, Jiménez-Anguiano A, Murillo-Rodríguez E, Prado-Alcalá RA (2000). Cortistatin modulates memory processes in rats. Brain Res.

[74] Hurst RD, Clark JB (1998). Alterations in transendothelial electrical resistance by vasoactive agonists and cyclic AMP in a blood–brain barrier model system. Neurochem Res.

[75] Lampugnani MG, Giorgi M, Gaboli M, Dejana E, Marchisio PC (1990). Endothelial cell motility, integrin receptor clustering, and microfilament organization are inhibited by agents that increase intracellular cAMP. Lab Invest.

[76] Dixit VD, Schaffer EM, Pyle RS, Collins GD, Sakthivel SK, Palaniappan R (2004). Ghrelin inhibits leptin- and activation-induced proinflammatory cytokine expression by human monocytes and T cells. J Clin Invest.

[77] Córdoba-Chacón J, Gahete MD, Culler MD, Castaño JP, Kineman RD, Luque RM (2012). Somatostatin dramatically stimulates growth hormone release from primate somatotrophs acting at low doses via somatostatin receptor 5 and cyclic AMP. J Neuroendocrinol.

[78] Benz F, Liebner S, Cader Z, Neuhaus W (2022). Structure and function of the blood–brain barrier (BBB). Physiology, pharmacology and pathology of the blood–brain barrier.

[79] Montagne A, Zhao Z, Zlokovic BV (2017). Alzheimer’s disease: a matter of blood–brain barrier dysfunction?. J Exp Med.

[80] Zlokovic BV, Griffin JH (2011). Cytoprotective protein C pathways and implications for stroke and neurological disorders. Trends Neurosci.

